# Spatiotemporal mapping reveals Ccl8^hi^ macrophages as key drivers of testicular inflammaging

**DOI:** 10.1002/ctm2.70527

**Published:** 2025-11-17

**Authors:** Yanping Huang, Jiahui Yao, Zhiqiang Zhang, Bin Ouyang, Jintao Zhuang, Xianshen Sha, Canhui Qu, Chengqiang Mo, Mujun Lu, Nanhe Lin, Xiangzhou Sun, Qiyun Yang, Yun Xie

**Affiliations:** ^1^ Department of Urology and Andrology Renji Hospital, Shanghai Jiao Tong University School of Medicine, Shanghai Institute of Andrology Shanghai China; ^2^ Zhongshan School of Medicine Sun Yat‐sen University Guangzhou China; ^3^ Department of Urology and Andrology The First Affiliated Hospital, Sun Yat‐sen University Guangzhou China; ^4^ Department of Andrology The Seventh Affiliated Hospital, Sun Yat‐sen University Shenzhen China; ^5^ Center for Reproductive Medicine Guangdong Women and Children Hospital Guangzhou China; ^6^ Department of Urology The First Affiliated Hospital of Jinan University Guangzhou China

**Keywords:** CCL8, inflammaging, spatiotemporal transcriptomics, testicular macrophages

## Abstract

**Background:**

Testicular macrophages (TMs) are key regulators of testicular immune privilege and endocrine function in the testis. However, their age‐related heterogeneity and role in testicular degeneration remain poorly characterized.

**Methods:**

We performed spatial transcriptomics and FACS‐enriched single‐cell RNA sequencing (scRNA‐seq) to characterize testicular macrophage heterogeneity across ageing. Key findings were validated through intratesticular injection of recombinant CCL8 protein, organotypic culture of seminiferous tubules, and immunofluorescence analysis.

**Results:**

Using spatial transcriptomics, we identified pronounced Leydig niche senescence in aged testes, mechanistically linked to TM‐mediated inflammatory remodelling. Coupling FACS‐enriched TM isolation with scRNA‐seq resolved seven transcriptionally distinct subpopulations, including ageing‐associated subsets (*Ccl8* ^hi^ and *Cxcl13* ^hi^). These subsets exhibited inflammatory signalling rewiring (e.g., CCL8‐CCR2 axis) and activation of senescence transcriptional regulators (*ASCL2*, *SPI1*, *CEBPB*, *JUNB*), with conserved mediators (*CCL8*, *TREM2*, *IL1β*, and *CXCL2*) across murine and human testes. Functional validation showed that intratesticular injection of recombinant CCL8 protein in 3‐month‐old mice recapitulated ageing phenotypes, such as germ cell apoptosis and steroidogenic decline.

**Conclusions:**

Our multi‐omics atlas highlights TM heterogeneity as a driver of testicular inflammaging and identifies *CCL8* as a conserved target for therapeutic interventions aimed at mitigating age‐associated male reproductive decline.

**Key points:**

1. Spatiotemporal multi‑omics establish inflammaging as a defining feature of testicular ageing.
2. A refined CD74‑based sorting strategy improves enrichment of testicular macrophages.
3. scRNA‐seq resolves seven TM subsets with age‑dependent shifts.
4. TM‑secreted inflammatory mediators‐especially CCL8‐drive testicular inflammaging.

## Graphical abstract

Spatiotemporal multi‐omics profiling establishes “inflammaging” — the age‐associated escalation of inflammatory signals — as a defining characteristic of testicular ageing. To deepen our investigation into the immune component of this process, we developed a refined sorting strategy based on the surface marker CD74, which significantly improves the enrichment of testicular macrophages (TMs). Applying high‐throughput single‐cell RNA sequencing, we comprehensively resolved the testicular immune landscape, identifying seven distinct TM subsets and revealing profound age‐dependent shifts in their composition and dynamics. Furthermore, we pinpointed that TM‐secreted inflammatory mediators, with the cytokine CCL8 being a particularly prominent driver, are central to orchestrating the testicular inflammaging program.

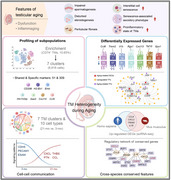

## 1 INTRODUCTION

Testicular macrophages (TMs), the predominant immune population within the testes, orchestrate both immune and non‐immune functions critical for testicular homeostasis, including immune privilege, phagocytosis, spermatogenesis, and steroidogenesis.^1^, ^2^, ^3^ Two major adult TM populations, the interstitial testicular macrophages (ITMs) and peritubular testicular macrophages (PTMs), have been identified based on their anatomical localization and function.^4^, ^5^, ^6^ ITMs, which coexist with Leydig cells (LCs), play a crucial role in their development, regeneration, and steroidogenesis.^5^, ^7^ PTMs, which surround the seminiferous tubules, are believed to regulate spermatogenesis.^7^ The polarized expression patterns of *CD115* (*CSF1R*) and major histocompatibility complex II (*MHCII*) align with their anatomical segregation: ITMs are *CSF1R*‐positive and MHCII‐negative, while PTMs are *CSF1R*‐negative and *MHCII*‐positive. Intriguingly, a *CSF1R* ^−^/*MHCII* ^−^ double‐negative subpopulation was detected, revealing an additional layer of TM heterogeneity beyond the canonical dichotomy.^5^ Macrophage heterogeneity exists across tissues; however, TMs remain poorly characterized, particularly in the context of ageing‐associated testicular decline.^8^, ^9^

Emerging characterization of macrophages using surface markers has unveiled underestimated heterogeneity in TMs.^10^ However, a comprehensive picture of the distinct macrophage subpopulations within the testis and their roles in natural ageing is yet to be fully understood with appropriate coverage and in‐depth characterization.^11^, ^12^, ^13^, ^14^ By utilizing advanced technologies such as single‐cell RNA sequencing (scRNA‐seq) and spatial transcriptomics (ST), we can better understand TM subsets and their roles in testicular homeostasis and ageing. Given the conservation of testicular immune privilege mechanisms in mammalian species, a cross‐species investigation is crucial to identify conserved ageing‐associated pathways in TMs. Recent multi‐organ transcriptomic analyses have identified *Ighm*, *C4b*, and *Ccl8* as consistently upregulated ageing‐associated biomarkers across diverse mouse organs.^15^ Ageing drives transcriptional reprogramming in diverse somatic cell types, leading to a shared pattern of immune activation.^16^ However, the contribution of ageing‐associated TM subsets to testicular endocrine and immune niche remodelling remains largely unknown, particularly how these cells drive chronic inflammation and fibrosis.^17^, ^18^, ^19^ These efforts to generate a detailed cellular and molecular atlas have been hampered by the scarcity of TMs and contamination from non‐meiotic and post‐meiotic germ cell types.^12^, ^20^, ^21^

Here, we present the first spatiotemporal profile of TMs during testicular ageing, combining CD74‐optimized fluorescence‐activated cell sorting (FACS) with high‐resolution scRNA‐seq (26 903 cells per age group) and 10x Visium‐based spatial transcriptomics. We resolved seven transcriptionally distinct TM subsets (2 872 TMs per age group), including ageing‐associated *Ccl8* ^hi^ and *Cxcl13* ^hi^ macrophages that drive inflammatory niche remodelling and Leydig cell decline. Functional studies leveraging in situ recombinant CCL8 protein injection demonstrated the detrimental role of TM‐derived chemokines in testicular fibrosis and endocrine decline. Overall, our findings establish a high‐resolution framework for TM profiling, identifying candidate biomarkers and therapeutic strategies for mitigating male reproductive ageing.

## 2 MATERIALS AND METHODS

### 2.1 Ethics approval statement

All experimental procedures involving animal experiments were approved by the Ethics Committee of the Sun Yat‐sen University Institutional Animal Care and Use Committee (SYSU‐IACUC‐2023‐001034).

### 2.2 Mice

All animal procedures conformed to the guidelines set by the International Council for Laboratory Animal Science. We used male wild‐type C57BL/6 mice aged approximately 3, 15, 21, and 27 months, sourced from the Experimental Animal Center of Sun Yat‐sen University (Guangzhou, China). The mice were housed in a specific pathogen‐free environment with a controlled 12 h light/dark cycle, temperature maintained at 20–26°C, and humidity at 50 ± 10%.

### 2.3 Histology and immunostaining

Testicular tissues were fixed in 4% paraformaldehyde buffered with PBS, then dehydrated and embedded in paraffin. Tissue sections of 5 µm thickness were prepared for hematoxylin and eosin (H&E) and Masson's trichrome (MT) staining. β‐galactosidase staining of frozen mouse testis and epididymis tissues was performed using the senescence‐associated β‐galactosidase (SA‐β‐gal) staining kit (Cat# G1073‐100T, Servicebio) following the manufacturer's protocol.

Paraffin‐embedded slides of mouse testis underwent deparaffinization in xylene, followed by rehydration through graded alcohol solutions. Endogenous peroxidase activity was blocked with 3% H_2_O_2_, and antigen retrieval was performed in 1× Tris‐EDTA buffer (pH 9.0, Cat# G1203, Servicebio). Tissues were permeabilized with 0.3% Triton‐X100 for 10 min, and non‐specific binding was blocked with 5% Bovine Serum Albumin for 30 min. Immunohistochemistry (IHC) was carried out on mouse testis sections using an Anti‐TNF‐alpha Rabbit pAb (1:100, GB11188, Servicebio), Anti‐CD68 Monoclonal Mouse antibody (1:100, sc‐20060, Santa Cruz), Anti‐CD74 Polyclonal Mouse antibody (1:100, sc‐6262, Santa Cruz), Anti‐CYP11A1 Polyclonal Rabbit antibody (1:100, 13363‐1‐AP, Proteintech Group, Inc.), Anti‐C‐C motif chemokine 8 Polyclonal Rabbit Antibody (1:100, A03237, Boster), with PBS as a negative control. Following PBS washes, sections were incubated at room temperature for 30 min with HRP Conjugated Goat Anti‐Rabbit IgG (1:100 dilution, Cat# CW0103, CWBIO) and subsequently counterstained with Hematoxylin Staining Solution (Cat# ZLI‐9610, ZSGB‐BIO). The Terminal deoxynucleotidyl transferase (TdT) dUTP nick‐end labelling (TUNEL) assay was conducted using the TUNEL apoptosis assay kit (HY‐K1091, MedChemExpress) in accordance with the manufacturer's protocol.

### 2.4 Visium slides and library preparation

Freshly collected testicular tissue was cleaned with dust‐free paper to remove surface residual liquid. The tissue was then embedded in optimal cutting temperature compound and rapidly frozen on dry ice. Embedded samples were stored at −80°C. Spatial transcriptome sequencing was performed using the 10x Genomics Visium platform with poly(A) capture for mRNA. A 10 µm tissue section was placed on the 10x Visium Spatial slide for methanol fixation, followed by H&E staining and image scanning, according to 10x Genomics' protocol. Tissue permeabilization, reverse transcription, cDNA amplification, and library construction were conducted using the 10x Genomics Visium Spatial Gene Expression Reagent Kit (PN‐1000184) and Slide Kit (PN‐1000185), following the manufacturer's guidelines. The resulting DNA libraries were sequenced in PE150 mode using high‐throughput sequencing.

### 2.5 10x Visium spatial RNA‐seq data preprocessing

The Space Ranger software pipeline (version 1.0.0) from 10x Genomics was utilized for processing Visium spatial RNA‐seq outputs and brightfield microscope images. This involved tissue detection, alignment of reads to the mm10 reference genome using the STAR aligner,^22^ generations of feature‐spot matrices, clustering, gene expression analysis, and spatial placement of spots on slide images. The resulting unique molecular identifier (UMI) count matrix. Data normalization was processed using Scanpy (v1.10.3).^23^ Mapping of tissue cell architecture was performed using integrated single‐cell and spatial transcriptomics analysis with Cell2location (v0.1.4).

### 2.6 Bulk RNA‐Seq

Total RNA was isolated from whole testes using the Qiagen RNeasy Mini Kit (Hilden), following the manufacturer's instructions. RNA‐seq libraries were prepared using the Illumina TruSeq Stranded Total RNA Library Prep Kit (Illumina Inc.). RNA quality and concentration were assessed with an Agilent 2100 Bioanalyzer (Agilent), combining microfluidics, capillary electrophoresis, and fluorescence. Electropherograms were produced by measuring the fluorescence intensities of 18S and 28S rRNA, and RNA integrity was evaluated using the RNA integrity number (RIN) algorithm, with all samples having RIN > 7 ensuring suitability for sequencing.

Sequencing was performed on an Illumina Novaseq 6000 platform (Illumina Inc.), generating 150 bp paired‐end reads. Raw reads in FASTQ format were processed using Fastp to remove low‐quality reads, resulting in clean reads. Alignment to the mouse reference genome (GRCm38/mm10) was conducted using Hisat2 (version 2.1.0). Read counts per gene were obtained with HTSeq‐count, and normalized as FPKM (fragments per kilobase of transcript per million mapped reads). Differential expression analysis was carried out using the DESeq2 R package, identifying significantly altered genes based on |log2FC| > 1.0 and FDR *p*‐value < .05.

### 2.7 Bulk RNA‐seq analysis

GO and pathway enrichment analyses were conducted using the ‘clusterProfiler’ R package.^24^ GSEA of GO biological processes was performed using the GSEA Java package (gsea2‐2.2.1.jar) from the Broad Institute.^25^ GO biological process annotations were sourced from org.Mm.eg.db (v3.12.0) for mouse. Significantly enriched GO terms were identified with an adjusted Benjamini–Hochberg *p*‐value < .05. Bioinformatic visualizations were created with OmicStudio tools (https://www.omicstudio.cn/tool).

### 2.8 1n‐depletion

Hoechst 33342 (Life Technologies) staining was performed on individual testicular cell suspensions as described in our previous publication.^21^ This method facilitates the identification and elimination of haploid germ cell subtypes, while retaining all other types of testicular cells.

### 2.9 Macrophage isolation from testis, CD74 staining and FACS

Testicular tissue was harvested from 3‐month‐old and 21‐month‐old mice. The tunica albuginea was removed, and the testicular tissue was minced to a paste‐like consistency. A digestion solution of 1 mL 1 mg/mL collagenase type IV (Sigma, C5138) was added, and the tissue was incubated at 37°C for 15 min with intermittent agitation. Digestion was halted by adding 6 mL of ice‐cold PBS, and the solution was filtered through a 70 µm mesh sieve. The filtrate was centrifuged at 1500 rpm for 5 min, and the supernatant was removed. The cell pellet was resuspended in approximately 1 mL PBS. Cells were stained with Alexa Fluor 647 anti‐mouse CD74 (<.5 µg/million cells) at 4°C for 30 min, diluted with 9 mL PBS, centrifuged again at 1500 rpm for 5 min, and the supernatant was discarded. The cells were resuspended in 2 mL ice‐cold PBS containing 2% FBS, mixed thoroughly, and filtered through a 40 µm sieve. Flow cytometry sorting was performed, and sorted cells were collected in a solution containing 1% bovine serum albumin (BSA).

### 2.10 Single‐cell RNA sequencing library construction

Cells were processed using the 10x Single Cell Controller according to the manufacturer's instructions. scRNA‐seq libraries were constructed with the Chromium Next GEM Single Cell 3′ v3.1 Library & Gel Bead Kit (10x Genomics) following the manufacturer's protocol. In summary, emulsions were created using the 10x Chromium Controller (10x Genomics) to target the recovery of 10 000 cells. Barcoded cDNAs were extracted from the emulsion and amplified by PCR (11 cycles). The cDNAs were then fragmented, end‐repaired, and A‐tailed before sample indexes were added via PCR (16 cycles). The final libraries were sequenced using the NovaSeq 6000 platform.

### 2.11 Single‐cell RNA sequencing data analysis

The gene‐barcode matrices were processed and analyzed using Scanpy (v1.10.3). For each sample, cells with a mitochondrial gene content exceeding 20% and a total gene count greater than 7000 or less than 200 were excluded from further analysis, selecting the top 2 000 variable genes. Batch effects across samples were corrected using Harmony. The resulting integrated Seurat object was normalized and processed through principal component analysis. For dimensional reduction, the top 30 principal components (PCs) were used in Uniform Manifold Approximation and Projection (UMAP) and for the construction of a K‐nearest neighbour graph. We identified clusters using the Leiden function with a resolution of  .5. Cluster‐specific markers were detected using the rank gene (Wilcoxon) function with the Wilcoxon rank sum test. Cell types were annotated based on known marker genes.^26^

We utilized CellChat^27^ to infer cell–cell communications from single‐cell gene expression data under preterm labour and control conditions. This analysis incorporated a database of known interactions between signalling ligands and receptors. Aggregated cell–cell communication patterns across different cell groups were computed for both study groups. We compared the interaction strengths among different cell types between 3‐ and 21‐month‐old mice, representing the differences in interaction strengths using heatmaps. Additionally, we identified major sending and receiving signalling roles for context‐specific pathways across distinct cell groups. The overall information flow between young and old groups was analyzed using the ‘rankNet’ function of CellChat.

### 2.12 Cell trajectory inference

Cellular differentiation trajectories were reconstructed using Palantir (v1.3.3) and VIA (v1.0.3). Monocle3 constructs the trajectory by utilizing the principal graph algorithm. VIA is a graph‐based trajectory inference algorithm that combines lazy‐teleporting random walks with Markov chain Monte Carlo refinement. It is implemented in the Python package pyVIA. The predicted cell trajectories from VIA and Monocle3 were visualized in the UMAP space, providing a comparison of the inferred developmental paths. Additionally, CytoTRACE2 (v1.1.0) was utilized to evaluate the differentiation state of the cells.

### 2.13 pySCENIC analysis

pySCENIC was employed to predict transcription factor regulatory networks.^28^ We utilized the normalized count matrix as input for SCENIC. To optimize computational efficiency, GRNBoost from the pySCENIC package was used in the initial step to infer potential transcription factor targets in all TMs, following default parameter settings for matrix expression filtering.

### 2.14 RNA isolation and quantitative PCR

Total RNA was extracted from tissues or seminal plasma using RNAiso Plus (Cat# 9109, Takara) according to the manufacturer's protocol. Two micrograms of total RNA were reverse transcribed into cDNA using the PrimeScript RT Master Mix Kit (Cat#RR036A, Takara) with incubation at 37°C for 15 min followed by 85°C for 5 s. Quantitative PCR (qPCR) was performed using TB Green Premix Ex Taq II (Cat# RR820A, Takara) following the manufacturer's instructions. The qPCR reactions were carried out on a LightCycler 480 II real‐time PCR system (Roche), initiated with denaturation at 95°C for 30 s, followed by 80 cycles of 95°C for 5 s and 60°C for 20 s. A melting curve analysis was conducted with steps at 95°C for 5 s, 60°C for 1 min, and 95°C for 5 s. Primer sequences used in the study are listed in Table S1 (Sangon Biotech).

### 2.15 Intratesticular injection of recombinant CCL8 protein

Recombinant CCL8 protein (Cat# 151004, Cat# 50181‐M24E) was administered via transcutaneous injection into one testis of anaesthetized mice at a concentration of 50 µg/100 µL, using a 27G needle. Control groups received 20 µL saline injections as a mock treatment. Animals were euthanized at 36 days postinjection (dpi) for analysis.

### 2.16 Johnsen's score

In assessing testicular tissue, we utilized Johnsen's scoring system, which employs a 10‐point scale to evaluate spermatogenesis based on the cellular composition within seminiferous tubules.^29^ A score of 10 represents optimal spermatogenic activity, whereas a score of 1 denotes a complete absence of germ cells.

### 2.17 Serum testosterone concentration assay

Testicular samples were collected at designated time intervals to quantitatively assess testosterone levels. Serum testosterone concentrations were measured using a commercially available ELISA kit (CEA458Ge, Cloud‐Clone Corp) following the manufacturer's instructions.

### 2.18 Isolation and organotypic culture of seminiferous tubules

The seminiferous tubule culture method for mice was adapted from established protocols.^30^ Under sterile conditions, seminiferous tubules were isolated from 3‐month‐old C57BL/6 mice and trimmed into uniform segments (∼5 cm). These tubule fragments were then transferred to 24‐well plates containing M199 medium, supplemented with  .1% BSA, 15 mM Hepes, 2.2 mg/mL sodium bicarbonate, 100 U/mL penicillin, 100 µg/mL streptomycin, and an insulin‐transferrin‐selenium (ITS) supplement. Cultures were maintained at 34°C under 5% CO_2_ conditions for 48 h per treatment group. For all experiments, seminiferous tubules were treated with either recombinant CCL8 protein or a normal control solution before being subjected to immunostaining for Uchl1 and CYP11A1. Apoptotic cells were detected using the One Step TUNEL Apoptosis Detection Kit (Cyanine 3) (HY‐K1079, MedChemExpress) according to the manufacturer's protocol. Fluorescence imaging was performed using a Zeiss LSM780 confocal microscope.

### 2.19 Statistics analysis

Statistical details, including tests performed and definitions for central tendency and variability, are provided in the figure legends where applicable. Data exclusion was based on quality filtering (as described earlier), and data stratification was conducted using Louvain clustering. Unless otherwise stated, all bar plots present data as mean ± SEM, while box plots show the first and third quartiles as the lower and upper bounds, with the median indicated by a thicker line inside the box; whiskers extend to 1.5 times the interquartile range. Gene expression differences were tested using likelihood ratio tests (via the DESeq2^31^ package in R), unless otherwise noted, and other comparisons were made using Student's *t*‐test. In cases where more than 10 tests were performed, *p*‐values were adjusted for false discovery. *p‐*values indicating statistical significance are denoted in the figures as follows: *p <  .05, **p <  .01, and ***p <  .001.

## 3 RESULTS

### 3.1 Spatiotemporal profiling links SASP‐Secreting TMs to Leydig niche senescence in ageing testes

**FIGURE 1:**
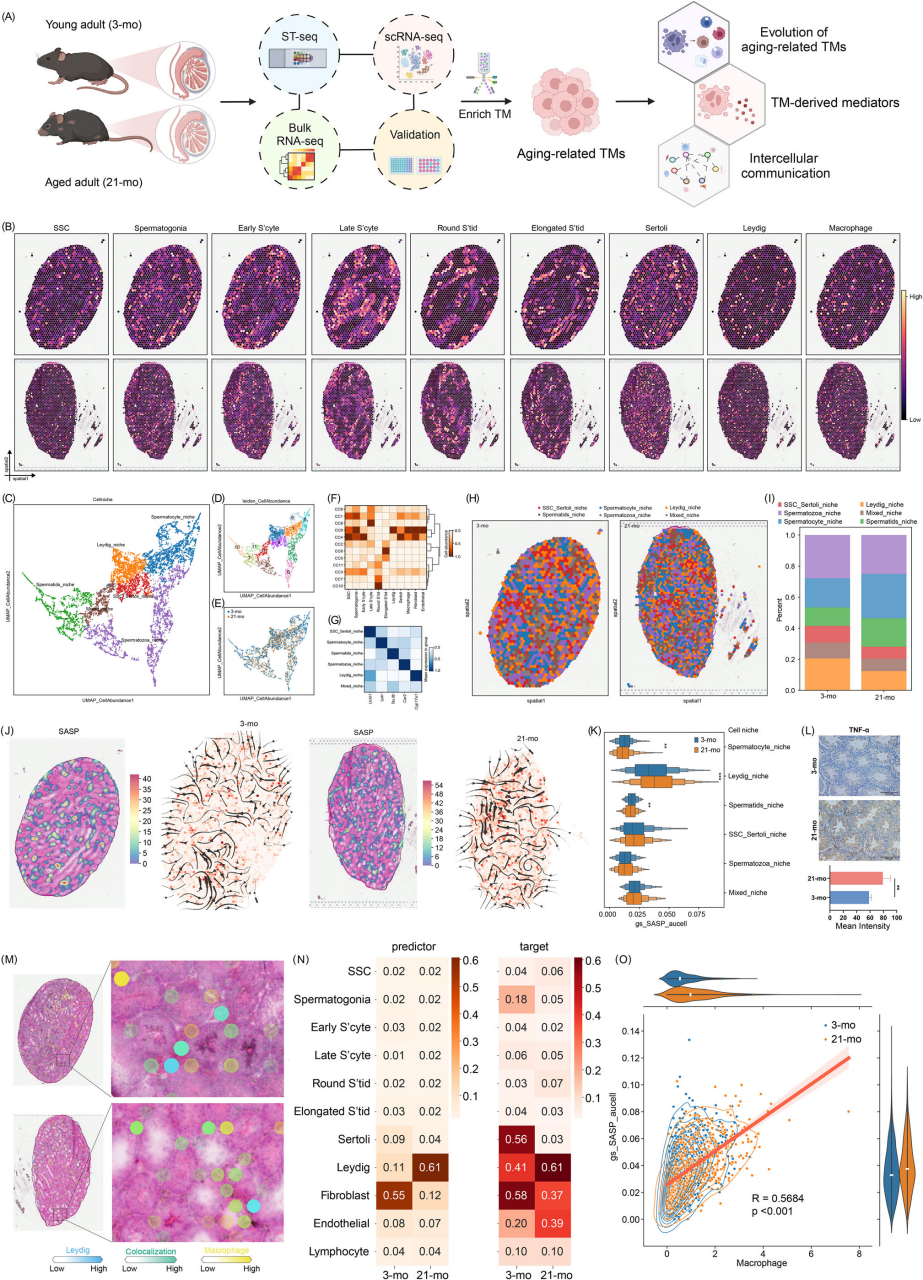
Spatial transcriptomics identifies interstitial senescence as a signature of age‐related testicular change. (A) Schematic of experimental workflow for decoding ageing‐related testicular macrophages in testes from young (3‐month‐old) and aged (21‐month‐old) mice. (B) Representative images of the 10X Genomics Visium maps for each age group, showing the expression patterns of cell‐type deconvolution, for the early spermatocytes, late spermatocytes, round spermatids, and elongating spermatids, respectively. (C–E) UMAP visualization of zonated gene expression (C), cell abundance (D) and samples of young and aged testes in 10X Genomics Visium datasets (E). (F, G) Heatmap demonstrating the cell distribution (F) and niche annotation (G). (H) Spatial mappings showing the testicular niches in young and aged testicular tissues. (I) Frequencies from Visum data of testicular niches from young and aged testes. (J) Expression of SASP genes and RNA velocity in young and aged testes. (K) The AUCell scores of the SASP genes of testicular niches. Statistical significance was determined using Student's *t*‐test. (L) Representative IHC images for TNF‐α in young and aged testes and quantification. *n* = 6 mice per group. Unpaired two‐tailed *t*‐test. Data are mean ± SD. (M) The spatial mappings showing the interstitial cells in young and aged testicular tissues, including macrophages and Leydig cells. (N) Cell–cell interactions in the Visum data. (O) Spearman's correlation between TMs and the activity score of SASP genes.

Using multi‐omics data, we sought to systematically investigate age‐related phenotypes, spatial heterogeneity, and transcriptomic differences in mouse testes during natural ageing (Figure 1A). Initially, we assessed the morphological features of the testes in C57BL/6 male mice at various stages (3, 15, 21, and 27 months of age) corresponding to human ageing (Figure S1A).^32^, ^33^, ^34^ The mice exhibited hair loss with age. Testicular histomorphometry showed that the relative testis weight was lowest at 21 months (Figure S1B,C). The seminiferous tubules exhibited evident alterations, including abnormal histological structures and decreased seminiferous epithelial thickness (Figure S1D). Consistently, our previous work demonstrated that 21‐month‐old C57BL/6 males exhibit systemic reproductive decline, including reduced epididymal sperm concentration and motility, impaired endocrine function, and marked testicular histological alterations, supporting their use as an aged model.^35^ Ageing was marked by increased SA‐β‐gal, interstitial fibrosis, and collagen deposition (Figure S1D).

To assemble a detailed spatial gene expression atlas of the mouse testes during ageing, we performed 10x Visium spatial transcriptomics analysis of fresh‐frozen samples, obtaining the transcriptional signatures of 8 464 spots, with a median of 2 116 spots per sample.^10^ Upon integration of annotated scRNA‐seq cell clusters with spatial transcriptomics spot clusters using a marker gene‐guided strategy (Figure 1B), we identified six major cell‐type niches: SSC‐Sertoli niche (4), spermatocyte niche (1, 8 and 9), spermatozoa niche (2 and 6), spermatids niche (7, 10, and 11), Leydig niche (0) and mixed niche (3) (Figure 1C–E). We observed strong dependencies between Leydig cells and macrophages, which were strongly co‐enriched in niche 0 (Figure 1F), in line with a known key role of macrophages in Leydig cell steroidogenesis.^36^ These cell type‐specific niches correspond well to the full spectrum of cell types identified in the scRNA‐seq data, as annotated by classical markers and reflected in cell‐type proportions (Figure 1G–I). Next, we checked gene sets related to the senescence‐associated secretory phenotype (SASP) in testicular niches (Figure 1J), as SASP is a hallmark of senescent cells.^37^, ^38^ To explore the molecular dynamics of SASP, we applied RNA velocity analysis (Figure 1J). Notably, the Leydig cell niche exhibited the highest SASP gene set score, which showed significant changes with ageing. Spatial visualization of these niches revealed that ageing led to a reduced proportion of the Leydig cell niche.^39^ Consistent with this, we observed elevated TNF‐α levels through IHC(Figure 1L). In line with previous reports and our SA‐β‐gal staining results, the Leydig cell niche was identified as the predominant senescent region in the testis.

To further investigate the regulatory mechanisms underlying this phenomenon, we analyzed the effects of colocalization and cell–cell interactions within the Leydig cell niche on gene expression states. Our findings revealed strong interdependencies between Leydig cells and macrophages, particularly in regions with high SASP expression (Figure 1M,N). Correlation analysis further demonstrated a strong association between macrophages and Leydig cells, indicating a close functional relationship within the ageing testicular microenvironment (Figure 1O). These findings underscore the pivotal role of TMs in orchestrating age‐associated testicular decline, with their abundance closely linked to SASP‐driven inflammation.

### 3.2 Refined sorting strategy to enrich TMs for single‐cell high‐resolution profiling

To elucidate the testicular cell populations intimately involved in inflammaging, we employed scRNA‐seq with 1n‐depletion to reduce the contamination of haploid germ cells (Figure S2A).^21^ We comprehensively profiled all the predominant cellular components in the testes of both young (3 months) and aged (21 months) mice (Figure S2B). Based on the expression of known markers, five somatic clusters were annotated as macrophages, LCs (*Insl3*), endothelial cells (*Cldn5*), fibroblasts/myoid cells (*Dcn*), and Sertoli cells (*Cst9*), and the four germ clusters were annotated as spermatogonia (*Uchl1*), spermatocytes (*Ccdc39*), round spermatids (*Tex36*), and elongated spermatids (*Spata32*), respectively (Figure S2C).^20^ Spermatogonia and spermatocytes exhibited pronounced population decline during testicular ageing (Figure S2D). Macrophages exhibited marked age‐related senescence accompanied by increased expression of inflammatory markers, including *TNF‐α* and *IL‐1β*, during testicular ageing (Figure S2E). Notably, even after minimizing the contamination with haploid sperm, a small number of enriched TMs remained. Nevertheless, analysis of previously published and our own single‐cell transcriptomic data from diverse murine cohorts revealed a paucity of TMs, which constrains a thorough understanding of their heterogeneity (Figure S3A,B).

Given the technical limitations stemming from the scarcity of TMs, we devised a new fluorescence‐activated cell sorting (FACS) strategy to gain further insight into TMs during ageing. We investigated a definitive panel of marker genes that defined macrophages using the CellMarker2.0 database (Figure S3C). *CD74* appeared to be an omnipresent surface marker of macrophages, with robust expression in both young and aged mice (Figure S3D,E). *CD74* expression specificity in TMs was confirmed by comparing the mean expression levels of this signature gene in scRNA‐seq and spatial transcriptomic data (Figure S3F,G).^40^, ^41^, ^42^ The Human Protein Atlas dataset revealed that CD74 was primarily expressed in macrophages and was strongly correlated with macrophage markers, suggesting that macrophages were the predominant source of CD74 (Figure S3H). At the bulk RNA level, old testes expressed a similar amount of *CD74* as young testes (Figure S3I). Consistently, quantitative PCR (qPCR) exhibited abundant expression of *CD74* in young and old testes (Figure S3J). This was supported by IHC staining of mouse testes at 3 months and 21 months (Figure S3K). Collectively, these findings support *CD74* as a robust pan‐macrophage marker in the testis, enabling efficient enrichment and downstream profiling of TMs during ageing.

### 3.3 Unsupervised classification reveals TM transcriptomic heterogeneity and functional diversity

**FIGURE 2:**
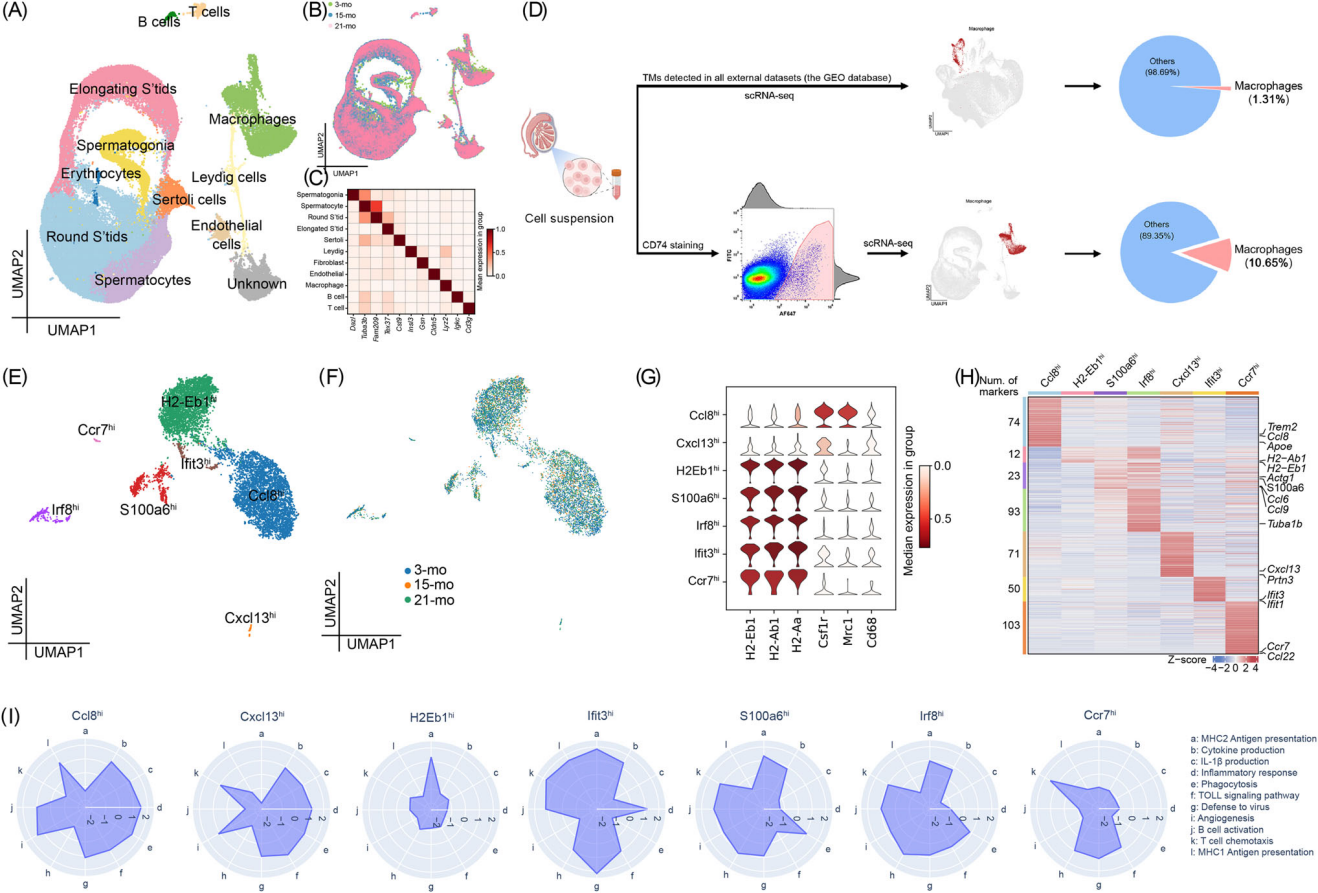
Unsupervised classification reveals TM transcriptomic heterogeneity and functional diversity. (A, B) UMAP plots of CD74^+^ sorted cells from 3‐, 15‐, and 21‐month‐old mice testes (*n* = 6–8 per age). (C) Heatmap of selected marker genes used for cell‐type annotation. (D) Schematic comparison of TM proportions using the previous method versus the CD74⁺ enrichment approach. (E, F) UMAP plots of TMs annotated by signature gene expression (E) and age (F). (G) Expression of key markers distinguishing peritubular and interstitial TMs. (H) Heatmap showing characteristic marker genes across TM subtypes. Colour indicates Z‐score–normalized expression. (I) Gene expression programs for each TM subset. Line lengths indicate the magnitude of transcriptional changes.

Next, we performed scRNA‐seq of FACS‐enriched CD74⁺ TMs from 3‐, 15‐, and 21‐month‐old mice (*n* = 6–8 per group) and identified subclusters using curated marker genes (Figure 2A–C). This CD74‐based gating strategy enabled us to recover an ∼8.1‐fold higher percentage of TMs compared with previous Hoechst‐based approaches (Figure 2D). After enrichment, we focused on the transcriptomic heterogeneity and functional roles of TMs. Reclustering the immune compartment revealed seven transcriptionally distinct TM subpopulations spanning the ageing process (Figure 2E,F).^43^ Clusters were annotated based on selective expression of marker genes (*Ccl8*, *Cxcl13*, *H2Eb1*, *S100a6*, *Irf8*, *Ifit3*, and *Ccr7*). Notably, Ccl8^hi^ and Cxcl13^hi^ macrophages expressed high levels of interstitial TM markers (Csf1r, Mrc1, Cd68), while other clusters preferentially expressed peritubular markers (*H2Eb1*, *H2Ab1*, and *H2Aa*) (Figure 2G).

Each TM subset exhibited distinct transcriptomic profiles (Figure 2H). To further characterize TM subpopulations, we computed gene signatures by selecting marker genes. GO term analysis revealed that H2Eb1^hi^ TMs were predominantly associated with antigen presentation, whereas *Ccl8* ^hi^ and *Cxcl13* ^hi^ TMs were significantly enriched for inflammatory response, IL‐1β production, and cytokine‐mediated signalling (Figure 2I). Additionally, *Ccl8* ^hi^ TMs, *Cxcl13* ^hi^ TMs, *S100a6* ^hi^ TMs, and *Tuba1b* ^hi^ TMs were strongly linked to angiogenesis, further emphasizing their potential roles in testicular immune remodelling. Overall, unsupervised clustering underscored the extensive functional heterogeneity of TMs, reflecting their diverse contributions to immune regulation and ageing‐associated inflammation.

### 3.4 Trajectory analysis identifies H2Eb1^hi^ TMs as the progenitors of macrophage lineages during testicular ageing

**FIGURE 3:**
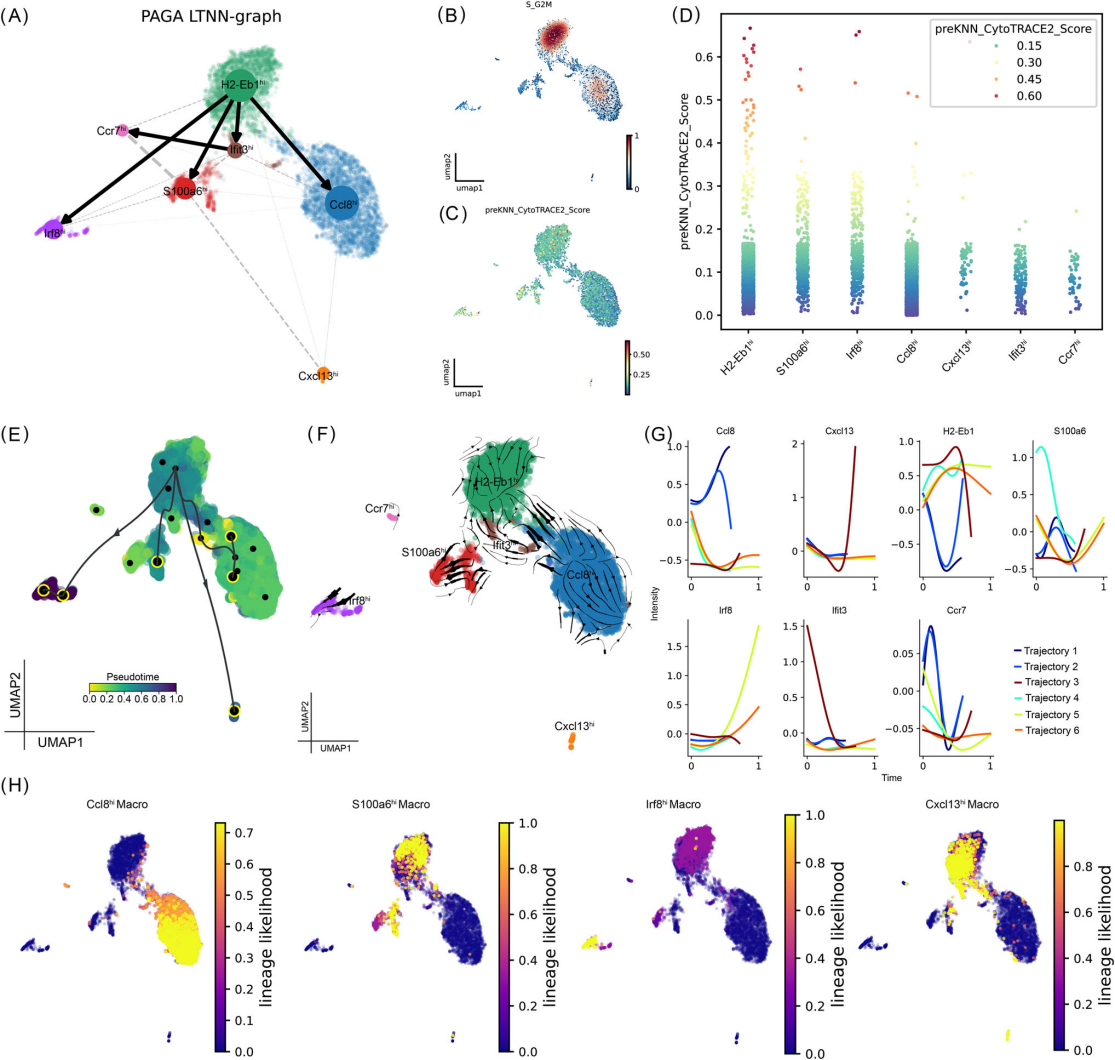
Trajectory analysis identifies H2Eb1⁺ TMs as the progenitors of macrophage lineages during testicular ageing. (A) PAGA trajectory visualization illustrating the differentiation pathways of TM subsets, with dot size proportional to the number of cells within each cluster. (B) Cell cycle scoring for TMs. (C) UMAP of TMs by differentiation state inferred by CytoTRACE. (D) Comparison of CytoTRACE scores for each TM subset. (E, F) UMAP plots displaying VIA pseudotime, with predicted TM cell fates highlighted in red‐black circles (E) and TM subset annotations (F). (G) Expression dynamics of TM markers along the differentiation trajectories. (H) UMAP plots annotated by major TM subsets.

To reveal the transcriptional transition of TMs during ageing, we traced the most likely origin of TMs by scoring cell‐cycle‐phase‐dependent gene expression and calculating the connections between clusters (Figure 3A). *H2Eb1* ^hi^ TMs exhibited the highest scores for S and G2/M phase markers (Figure 3B,C), suggesting a proliferative and potentially progenitor‐like state. CytoTRACE analysis further indicated that *H2Eb1* ^hi^ TMs possessed the greatest differentiation potential (Figure 3D). We validated the sequential trajectory sprouting from the *H2Eb1* ^hi^ TMs using trajectory inference methods based on the distinct algorithmic principle of VIA (Figure 3E,F). We observed that *Ccl8*, *Cxcl13*, and *Irf8* were significantly upregulated at the terminal stage of the trajectory (Figure 3G). We further identified signature expression patterns across distinct TM lineages (Figure 3H). These findings indicate that *H2‐Eb1* ^hi^ TMs function as the progenitor population giving rise to distinct macrophage lineages, including *Ccl8* ^hi^, *Cxcl13* ^hi^, and *Irf8* ^hi^ TMs.

### 3.5 Ageing reshapes macrophage‐mediated intercellular communication and hyperactivates CCL8 signalling

**FIGURE 4:**
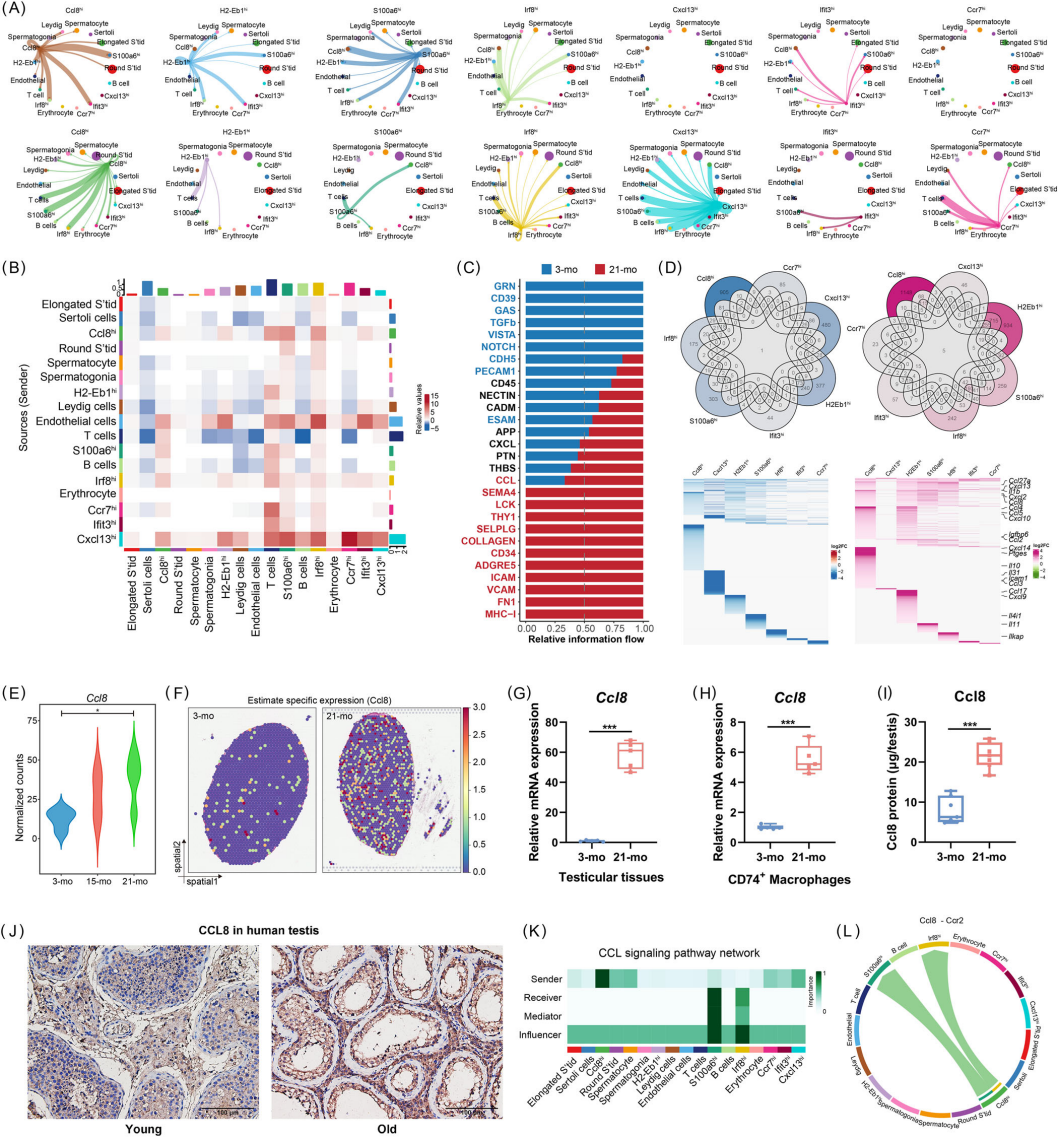
Ageing reshapes macrophage‐mediated intercellular communication and hyperactivates CCL8 signalling. (A) Circle plots showing communication and interaction strength in each cell cluster of testes from 3‐ and 21‐month‐old mice. (B) Heatmap showing differential interaction strength in each testicular cell type between 3‐ and 21‐month‐old mice. (C) The significant signalling pathways of TMs ranked based on the inferred strength differences between 3‐ and 21‐month‐old mice. (D) Overlap of TM subsets gene expression and profiles in young versus aged testes. (E) Gene expression level of *CCL* in bulk RNA‐seq data of the testes from 3‐ and 21‐month‐old mice (*n* = 4). (F) Gene expression levels of *CCL8* based on ST‐seq data from 3‐month‐old and 21‐month‐old mice testes. (G–I) qRT‐PCR analyses of *CCL* expression in testicular tissue (G) and CD74^+^ TMs (H), and ELISA of CCL8 in testicular interstitial fluid (I) (*n* = 6). (J) IHC for CCL8 expression in human testes. (K, L) Heatmap of CCL signalling pathways within cellular clusters (K) and chord diagram of *Ccl8*‐*Ccr2* ligand‐receptor interactions (L).

Having established the testicular cell types most affected by ageing, we sought to quantitatively infer and analyze the intercellular communication networks.^27^ As an integrative hallmark of ageing, altered intercellular communication was evident in aged testicular tissues (21‐month‐old) compared with young testicular tissues (Figure 4A,B).^44^, ^45^ Next, we identified the signalling pathways involved in TM communication during ageing (Figure 4C). We compared the communication probabilities of cell–cell clusters in the signalling pathways that participate in TM communication with advancing age, including CCL, THBS, CD45, and ICAM.

To delineate age‐related transcriptional dynamics in TMs, we performed differential expression analysis comparing macrophage populations from young versus aged murine testes (Figure 4D). Of note, the *Ccl8* ^hi^ subpopulation demonstrates the most pronounced differential expression profile. Longitudinal bulk RNA‐seq analysis of the *Ccl8* signature across three age cohorts (3, 15, and 21 months) revealed progressive upregulation of Ccl8 expression correlating with advancing age (Figure 4E). Spatial transcriptomic profiling confirmed elevated *Ccl8* expression patterns in aged testicular tissue compared with young controls (Figure 4F). This age‐dependent elevation was consistently validated through three orthogonal approaches: (1) qRT‐PCR quantification in both whole testicular tissue and CD74^+^ TMs (*p* < .01), (2) protein‐level detection via ELISA in testicular interstitial fluid (1.8‐fold increase, *p* = .003), and (3) immunohistochemical analysis demonstrating significantly enhanced CCL8 immunoreactivity in aged human testicular specimens compared with younger counterparts (Figure 4G–J). Consistent with the enhanced cytokine‐mediated signalling in aged testes, *CCL8*‐*CCR2* was prominent in the CCL signalling of testicular intercellular interactions (Figure 4K,L). The concordant transcriptional upregulation, spatial distribution patterns, and protein‐level validation across murine and human testes not only establish CCL8 as a conserved biomarker of testicular ageing but also implicate its functional involvement in orchestrating immune cell recruitment and intercellular communication within the senescent testicular niche.

### 3.6 Conserved proinflammatory signatures in ageing testicular macrophages across humans and mice

**FIGURE 5:**
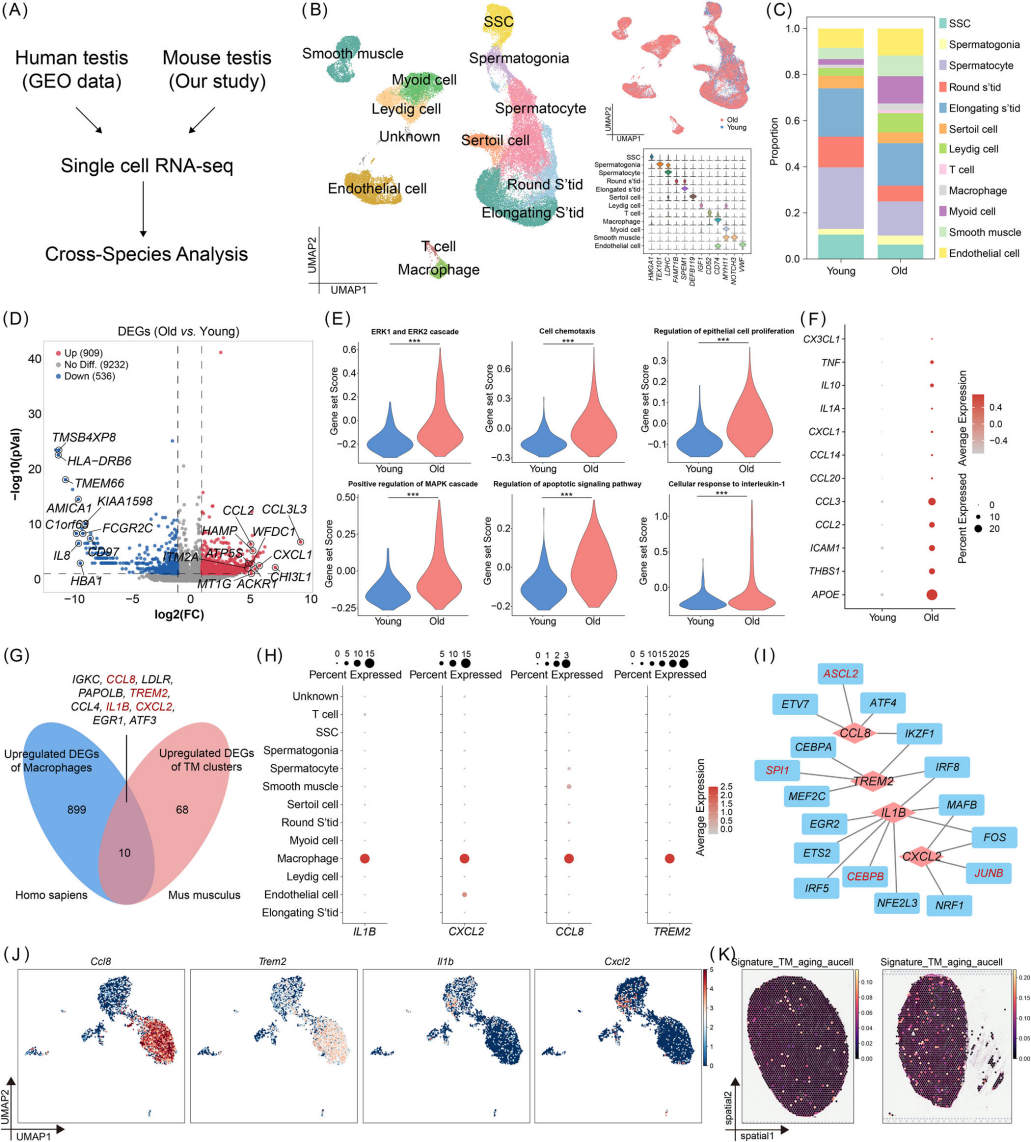
Conserved proinflammatory signatures in ageing testicular macrophages across humans and mice. (A) Overview of the data collection and analysis workflow. Human testis GEO datasets: GSE182786, GSE142585, and GSE215754. (B) UMAP showing scRNA‐seq data of young and old human testes and cell type annotations. (C) Frequencies from scRNA‐seq data of testicular cells from the young and the old. (D) Volcano plot showing DEGs between young and old human TMs. (E) Gene set scores comparing young and old human TMs. (F) Dot plot displaying selected ageing‐related genes. (G) Venn diagram of upregulated genes of TMs during ageing between human and mouse datasets. (H) Dot plots showing TM‐specific genes of human and mice during ageing. (I) Gene regulatory network of conserved ageing‐related genes and putative TFs. TFs shared with the Ageing Atlas database are highlighted in red. (J) UMAP feature plots of key markers (CCL8, TREM2, IL1β, CXCL2) expression in TM‐enriched scRNA‐seq data. (K) Spatial distribution of the ageing score, defined by four key marker genes in (J).

To further investigate the transcriptional changes associated with advancing age in human TMs, we performed a joint analysis using human testicular scRNA databases (Figure 5A). Cell‐type annotation was conducted based on classical marker genes (Figure 5B). A significant increase in the proportion of macrophages was observed in the old group (Figure 5C). Furthermore, differential expression analysis of TMs showed significant upregulation in the expression of chemokines, such as *CCL3*, *CCL2*, and *CXCL1* (Figure 5D). Accordingly, *ASCL2*, *JUNB*, *SPI1*, and *CEBPB* were identified as key cell‐specific transcription factors (TFs) in human TMs during ageing (Figure S4A–C).^46^ Gene set scores of these upregulated genes highlighted the significant activation of pathways, including the ERK1 and ERK2 cascades, cell chemotaxis, and the MAPK cascade (Figure 5E). Furthermore, the chemotaxis‐related pathways showed a striking upregulation of multiple signalling mediators in aged individuals (Figure 5F). Notably, by intersecting the differential expression genes (DEGs) in human TMs with our enriched macrophage data from the murine model of natural ageing, we identified 10 shared genes, of which *IL‐1β*, *CXCL2*, *CCL8*, and *TREM2* were macrophage‐specific ageing‐related genes (Figure 5G,H). We identified gene regulatory networks and spatial expression profiles of these key DEGs using SCENIC analysis (Figure 5I,J). Additionally, senescence scoring of TMs revealed an elevated senescence signature in aged testicular macrophages (Figure 5K). Collectively, these findings demonstrate that proinflammatory activation of TMs is a conserved hallmark of testicular ageing across mice and humans.

### 3.7 CCL8 triggers testicular inflammation and functional decline, recapitulating ageing‐related phenotypes

**FIGURE 6:**
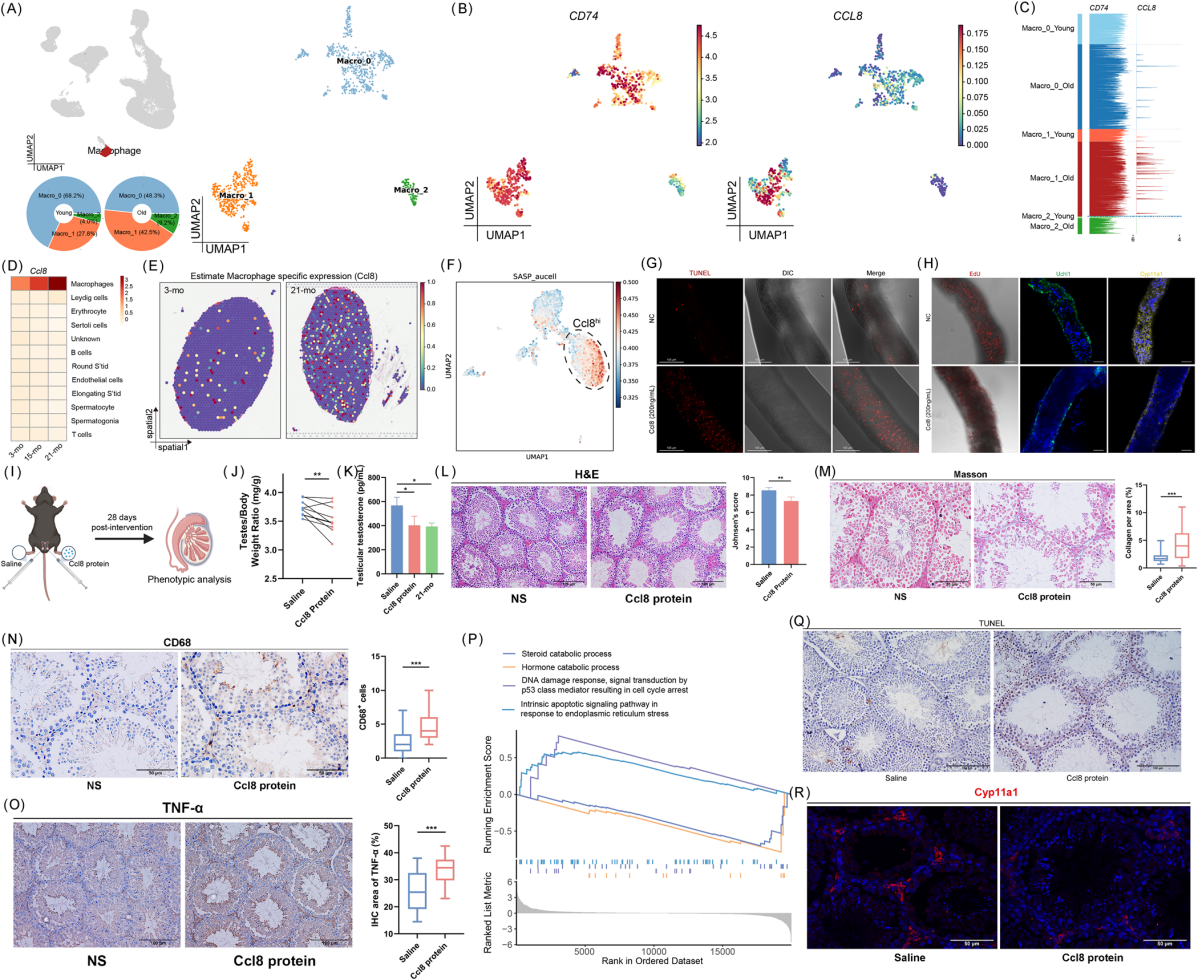
CCL8 triggers testicular inflammation and functional decline, recapitulating ageing‐related phenotypes. (A) UMAP of integrated scRNA‐seq data of macrophages (upper left), subpopulations (right) and cell proportions (lower left) in young and old human testis GEO datasets. Macro, macrophages. (B) UMAP of gene expression of *CD74* (left) and *CCL8* (right)in the human testicular scRNA‐seq datasets. (C) scRNA analysis of *CCL8* and *CD74* expression in macrophages from young and old human testes. (D) Heatmap illustrating *CCL8* expression in different testicular cell types from 3‐, 15‐, and 21‐month‐old mice. (E) Gene expression levels of *CCL8* based on ST‐seq data from 3‐ and 21‐month‐old mice. (F) Activity score of SASP gene signatures in *Ccl8*⁺ TMs. (G, H) Immunofluorescence staining of seminiferous tubules for TUNEL (G), EdU, UCHL1, and CYP11A1 (H) after 48 h of ex vivo culture with or without recombinant CCL8. (I) Schematic of mouse intratesticular injection. (J) Bar plots of testis‐to‐body weight ratio after testicular injection of saline or recombinant CCL8 protein in 3‐month‐old mice (*n* = 10). (K) Testosterone levels in the testes of 3‐month‐old and 21‐month‐old mice after CCL8 or saline injection (*n* = 3). (L) H&E staining and quantification of Johnsen's score of seminiferous tubules in the testes of 3‐month‐old mice after injection with saline or recombinant CCL8 protein (*n* = 6). (M) Representative images and quantification of Masson's trichrome staining to assess fibrosis in the testes of 3‐month‐old mice after injection with saline or recombinant CCL8 protein (*n* = 6). (N, O) Representative images and quantification of IHC of CD68 (N) and TNF‐α (O) expression in the testes of 3‐month‐old mice after treatment (*n* = 6). (P) GSEA of bulk RNA‐seq from testes of 3‐month‐old mice injected with saline or recombinant CCL8 (*n* = 6). (Q) Representative images and quantification of TUNEL staining in testes post‐injection (*n* = 6). (R) Immunofluorescence staining of Cyp11a1 in interstitial cells from testes following CCL8 or saline injection.

Given the high expression of chemokines derived from ageing‐associated macrophages, we hypothesized that these chemotactic proteins might induce testicular inflammation. Unsupervised clustering identified three macrophage subclusters—Macro0, Macro1, and Macro2—based on public human testicular datasets (Figure 6A). As expected, *CD74* was broadly expressed across human testicular macrophages, while *CCL8* was specifically enriched in the Macro1 subset, representing an ageing‐associated macrophage population with elevated expression during testicular ageing (Figure 6B,C). Single‐cell transcriptional profiling of macrophage‐specific chemokines revealed distinct cluster‐specific expression patterns, with Ccl8 emerging as a significantly upregulated chemokine during testicular ageing (Figure 6D,E). Transcriptional profiling of SASP components demonstrated preferential enrichment in Ccl8^hi^ macrophages compared with other TM subsets (Figure 6F), suggesting its pivotal role in initiating age‐related inflammatory responses.

Immunofluorescence analysis of cultured seminiferous tubules revealed reduced proliferation, increased apoptosis, and disrupted expression of UCHL1 and CYP11A1 (Figure 6G,H). To evaluate the functional consequences of CCL8 signalling in vivo, we performed intra‐testicular injections of recombinant CCL8 protein or saline in 3‐month‐old mice (Figure 6I). Mice treated with CCL8 exhibited a significant reduction in testis‐to‐body weight ratio, lower Johnsen scores, and increased collagen deposition—hallmarks of testicular fibrosis (Figure 6J–M). IHC analysis revealed increased CD68⁺ macrophages, indicative of inflammatory infiltration (Figure 6N), and elevated TNF‐α expression, a classical SASP component (Figure 6O). Bulk RNA‐seq followed by GSEA revealed significant upregulation of cell cycle arrest and apoptotic signalling pathways, along with downregulation of steroid catabolic processes in CCL8‐treated testes (Figure 6P). Germ cells in the CCL8‐treated group exhibited a higher number of TUNEL‐positive signals compared with the saline‐injected group, suggesting that CCL8 promotes germ cell apoptosis (Figure 6Q). Finally, CYP11A1 expression was markedly diminished in testicular interstitial cells in vivo (Figure 6R), indicating impaired testosterone biosynthesis. Collectively, these results demonstrate that CCL8 induces testicular inflammation, germ cell loss, fibrosis, and endocrine dysfunction—phenotypes that recapitulate key features of natural ageing.

## 4 DISCUSSION

Macrophages are innate immune cells that exhibit a myriad of functional properties tailored to the specific environment of nearly all mammalian organs. These cells represent the predominant immune cell population in the murine testicular interstitium.^10^ However, the evidence regarding the heterogeneity of TMs during ageing is limited. In this study, we employed state‐of‐the‐art single‐cell sequencing technologies, including scRNA‐seq with enrichment and spatial transcriptomics, to construct a comprehensive high‐resolution transcriptomic atlas of TMs. This dataset captures dynamic, age‐dependent transcriptional changes from early adulthood to late ageing, providing a spatiotemporal map of TM heterogeneity.

Our data revealed that macrophages exhibited stronger age‐related expression changes in subpopulations with an inflammatory phenotype than in those with an immunosuppressive phenotype. Increasing expression of proinflammatory cytokines (e.g., *IL1β*, *TNF‐α*, etc.) and senescence‐specific marker (*Cdkn1a*) were mainly observed in aged TMs at the single‐cell level.^47^, ^48^ This is consistent with previous studies showing that inflammation is a hallmark of age‐related testicular dysfunction.^49^, ^50^ Recent spatial transcriptomic analyses of testicular tissue have shown that Leydig cells are major senescence‐sensitive spots.^51^ Our own spatial transcriptomic data also indicated significant ageing of the interstitial Leydig cell niche, and further findings suggest that macrophages are closely associated with the Leydig niche ageing. These results underscore the critical role of macrophages in testicular ageing.

Previous studies have struggled to understand the behaviour of distinct macrophage populations during testicular ageing, likely because of limited characterization, as TMs are scarce.^11^, ^12^, ^13^, ^14^ By advancing the general testicular cell profiling methods previously used in the field, the pan‐macrophagic marker CD74 allowed for the refined analysis of macrophage subpopulations.^21^ Consistent with our findings, CD74 was identified as a highly expressed marker gene of TMs in both human and murine testicular single‐cell transcriptomic datasets.^20^, ^52^ scRNA‐seq data of sorted TMs allowed us to identify subsets of TMs with an 8.1 times higher percentage of TMs among the total testicular cells compared with the previous gating strategy. The successful application of CD74 as a robust enrichment marker significantly enhances the toolkit for testicular immunology research.

Resident tissue macrophages comprise a heterogeneous population of immune cells that occupy multiple tissue niches and exhibit microenvironment‐specific phenotypes and functions.^8^ Building upon prior dichotomous classifications, our analysis provided a more refined and comprehensive characterization of the heterogeneity and dynamic changes within these TM populations during ageing.^10^ Notably, trajectory inference algorithms consistently identified H2Eb1^hi^ TMs as the primitive TM population, serving as a progenitor‐like subset. Together, these findings present the first high‐resolution transcriptional atlas of TMs during natural ageing in mice, offering novel insights into their diversity, lineage dynamics, and functional adaptations.

Many fundamental questions remain regarding TM subpopulations, cell–cell interactions, and TFs.^10^ Our data emphasize the diverse spatiotemporal functional states and enhanced cell–cell communication among macrophages across young and old age groups. The identification of seven TM subpopulations highlights the complexity of macrophage‐mediated processes in the ageing testicular microenvironment. Among these, H2Eb1^hi^ TMs exhibited a conserved antigen presentation program with minimal cytokine production, suggesting their role in immune surveillance. In contrast, CCL8^hi^ and CXCL13^hi^ TMs were significantly enriched for genes related to inflammatory cytokine production, including IL‐1β, TNF, and chemokine‐mediated signalling, indicating their strong inflammatory potential.^4^, ^53^ Trajectory analysis revealed a transition from H2Eb1^hi^ TMs to CCL8^hi^ and CXCL13^hi^ TMs during ageing, indicating a shift in macrophage function over time. Additionally, computational analyses identified a set of age‐associated TFs governing this transition. Among them, GATA6 was predicted as a key age‐related TF influencing the differentiation and functional remodelling of CXCL13^hi^ TMs.^54^ Notably, cytokine‐producing signatures were enriched in aged TM subsets, suggesting that age‐associated TMs exploit these pathways to sustain an inflammatory testicular microenvironment. To translate findings from mice to the study of testicular ageing in humans, we identified a set of conserved ageing‐related genes (e.g., *CCL8*, *TREM2*, *IL1β*, *CXCL2*, etc.) and potential TFs (e.g., *ASCL2*, *SPI1*, *CEBPB*, and *JUNB*) in TMs. Taken together, our study provides a high‐resolution map of TM heterogeneity, refines previous classification frameworks, and identifies novel subpopulations and TFs involved in testicular ageing.

Our findings have potential implications for understanding human age‐related male infertility. Advanced paternal age is associated with a decline in sperm quality, increased DNA fragmentation, and altered hormone profiles, which have been linked to changes in the testicular immune microenvironment. The identification of Ccl8^hi^ macrophages as a driver of testicular inflammaging suggests a mechanistic link between chronic inflammation and age‐associated spermatogenic decline. These results provide a foundation for developing novel biomarkers and targeted interventions aimed at mitigating inflammation‐driven testicular dysfunction in ageing men.

The datasets we provide offer a valuable resource for the understanding of the heterogeneity and evolutionary changes in TMs during ageing; however, this study has inherent limitations. First, although CD74‐based sorting markedly increases TM recovery compared with prior strategies, it is not an absolute solution because CD74 is not entirely macrophage‐specific. Our validation across scRNA‐seq, spatial transcriptomics, bulk RNA, qPCR, and IHC supports its utility for TM enrichment, yet future work will implement combinatorial gating together with explicit exclusion gates to further enhance specificity and purity. Second, direct observation of cell types, differential gene expression, and intercellular communication in age‐specific murine testes provides critical insights into ageing mechanisms. However, experimental interventions are necessary to establish causality in our findings. Third, the role of specific TFs in regulating DNA accessibility in ageing testicular cells remains challenging because of the difficulty in detecting low‐abundance transcripts such as TFs in scRNA‐seq data. Our functional assays following intratesticular CCL8 injection focused on testicular microenvironmental changes, and a comprehensive assessment of systemic fertility outcomes (e.g., epididymal sperm parameters and mating performance) will require genetic models and long‐term studies in future work. This limitation often results in technical artefacts known as dropouts, leading to potential false negatives. We recommend further investigation into the precise distribution and epigenomic characteristics of TM cell types because baseline differences in abundance could influence the observed ageing effects.

## 5 CONCLUSION

In this study, we present a high‐resolution, single‐cell spatiotemporal map of testicular macrophage (TM) heterogeneity during ageing, enabled by a CD74‐based enrichment strategy. Our integrative approach uncovered seven transcriptionally distinct TM subpopulations, among which Ccl8^hi^ macrophages emerged as key drivers of testicular inflammaging. These ageing‐associated TMs exhibited robust inflammatory signatures and were closely linked to Leydig niche senescence and testicular functional decline. The identification of Ccl8^hi^ TMs as key mediators of immune remodelling highlights new biomarkers and therapeutic targets. Together, our findings offer mechanistic insights into the immune dynamics of reproductive ageing and provide a valuable resource for developing strategies to mitigate age‐related testicular dysfunction.

## AUTHOR CONTRIBUTIONS

Conceptualization: Yun Xie, Yanping Huang, and Qiyun Yang; methodology: Jiahui Yao, Zhiqiang Zhang, Xianshen Sha, and Canhui Qu; validation: Jiahui Yao and Nanhe Lin; formal analysis: Zhiqiang Zhang and Nanhe Lin; data curation: Jintao Zhuang, Xianshen Sha, and Chengqiang Mo; writing—original draft: Zhiqiang Zhang and Canhui Qu; writing—review & editing: Yanping Huang, Bin Ouyang, and Qiyun Yang; visualization: Nanhe Lin and Jiahui Yao; supervision: Yanping Huang, Mujun Lu, Qiyun Yang, and Jintao Zhuang; project administration, Qiyun Yang and Xiangzhou Sun; funding acquisition: Yanping Huang, Jintao Zhuang, Qiyun Yang, Nanhe Lin, Xiangzhou Sun, and Yun Xie.

## CONFLICT OF INTEREST STATEMENT

The authors declare no conflict of interest.

## ETHICS STATEMENT

All experimental procedures involving animal experiments were approved by the Ethics Committee of the Sun Yat‐sen University Institutional Animal Care and Use Committee (SYSU‐IACUC‐2023‐001034).

## Supporting information



Supporting Information

Supporting Information

## Data Availability

The data underlying this article will be shared on reasonable request to the corresponding author. Additional datasets referenced in this study are available in the GEO database under accession numbers GSE182786, GSE142585, and GSE215754.

## References

[1] Zhao S , Zhu W , Xue S , Han D . Testicular defense systems: immune privilege and innate immunity. Cell Mol Immunol. 2014;11(5):428‐437. doi:10.1038/cmi.2014.38 24954222 PMC4197207

[2] Mossadegh‐Keller N , Sieweke MH . Testicular macrophages: guardians of fertility. Cell Immunol. 2018;330:120‐125. doi:10.1016/j.cellimm.2018.03.009 29650243

[3] Gu X , Li SY , Matsuyama S , DeFalco T . Immune cells as critical regulators of steroidogenesis in the testis and beyond. Front Endocrinol (Lausanne). 2022;13:894437. doi:10.3389/fendo.2022.894437 35573990 PMC9096076

[4] Mossadegh‐Keller N , Gentek R , Gimenez G , Bigot S , Mailfert S , Sieweke MH . Developmental origin and maintenance of distinct testicular macrophage populations. J Exp Med. 2017;214(10):2829‐2841. doi:10.1084/jem.20170829 28784628 PMC5626405

[5] DeFalco T , Potter SJ , Williams AV , Waller B , Kan MJ , Capel B . Macrophages contribute to the spermatogonial niche in the adult testis. Cell Rep. 2015;12(7):1107‐1119. doi:10.1016/j.celrep.2015.07.015 26257171 PMC4545310

[6] Gayer FA , Reichardt SD , Bohnenberger H , Engelke M , Reichardt HM . Characterization of testicular macrophage subpopulations in mice. Immunol Lett. 2022;243:44‐52. doi:10.1016/j.imlet.2022.02.003 35149127

[7] Meinhardt A , Wang M , Schulz C , Bhushan S . Microenvironmental signals govern the cellular identity of testicular macrophages. J Leukoc Biol. 2018;104(4):757‐766. doi:10.1002/jlb.3mr0318-086rr 30265772

[8] Chakarov S , Lim HY , Tan L , et al. Two distinct interstitial macrophage populations coexist across tissues in specific subtissular niches. Science. 2019;363(6432):eaau0964. doi:10.1126/science.aau0964 30872492

[9] Jain A , Gyori BM , Hakim S , et al. Nociceptor‐immune interactomes reveal insult‐specific immune signatures of pain. Nat Immunol. 2024;25(7):1296‐1305. doi:10.1038/s41590-024-01857-2 38806708 PMC11224023

[10] Meinhardt A , Dejucq‐Rainsford N , Bhushan S . Testicular macrophages: development and function in health and disease. Trends Immunol. 2022;43(1):51‐62. doi:10.1016/j.it.2021.11.003 34848166

[11] Mass E , Nimmerjahn F , Kierdorf K , Schlitzer A . Tissue‐specific macrophages: how they develop and choreograph tissue biology. Nat Rev Immunol. 2023;23(9):563‐579. doi:10.1038/s41577-023-00848-y 36922638 PMC10017071

[12] Zhang W , Xia S , Xiao W , et al. A single‐cell transcriptomic landscape of mouse testicular aging. J Adv Res. 2023;53:219‐234. doi:10.1016/j.jare.2022.12.007 36528294 PMC10658307

[13] Alfano M , Tascini AS , Pederzoli F , et al. Aging, inflammation and DNA damage in the somatic testicular niche with idiopathic germ cell aplasia. Nat Commun. 2021;12(1):5205. doi:10.1038/s41467-021-25544-0 34471128 PMC8410861

[14] Nie X , Munyoki SK , Sukhwani M , et al. Single‐cell analysis of human testis aging and correlation with elevated body mass index. Dev Cell. 2022;57(9):1160‐1176.e5. doi:10.1016/j.devcel.2022.04.004 35504286 PMC9090997

[15] FQ Y , XY W , YX L , et al.—Multiorgan transcriptomics in mice identifies immunoglobulin heavy constant mu. *D ‐ 7505876*. (‐ 1091‐6490 (Electronic)):‐ e2423142122. 10.1073/pnas.2423142122 PMC12280941 40643973

[16] Cui LA‐O , Nie X , Guo YA‐O , et al. Single‐cell transcriptomic atlas of the human testis across the reproductive lifespan. (2662‐8465 (Electronic)). 10.1038/s43587-025-00824-2 PMC12003174 40033047

[17] L C , Id O , X N , et al.—Single‐cell transcriptomic atlas of the human testis across the reproductive. *D ‐ 101773306*. (‐ 2662‐8465 (Electronic)):‐ 658‐674.

[18] L F , E F , —Inflammageing: chronic inflammation in ageing, cardiovascular disease. *D ‐ 101500075*. (‐ 1759‐5010 (Electronic)):‐ 505‐522. 10.1038/s41569-018-0064-2 PMC6146930 30065258

[19] A K , Id‐ Orcid X.—Multi‐omics perspectives on testicular aging: unraveling germline dysregulation. *D ‐ 101600052*. (‐ 2073‐4409 (Electronic)):T—epublish.

[20] Green CD , Ma Q , Manske GL , et al. A comprehensive roadmap of murine spermatogenesis defined by single‐cell RNA‐Seq. Dev Cell. 2018;46(5):651‐667.e10. doi:10.1016/j.devcel.2018.07.025 30146481 PMC6713459

[21] Gaysinskaya V , Soh IY , van der Heijden GW , Bortvin A . Optimized flow cytometry isolation of murine spermatocytes. Cytometry A. 2014;85(6):556‐565. doi:10.1002/cyto.a.22463 24664803 PMC4246648

[22] Dobin A , Davis CA , Schlesinger F , et al. STAR: ultrafast universal RNA‐seq aligner. Bioinformatics. 2013;29(1):15‐21. doi:10.1093/bioinformatics/bts635 23104886 PMC3530905

[23] Stuart T , Butler A , Hoffman P , et al. Comprehensive integration of single‐cell data. Cell. 2019;177(7):1888‐1902.e21. doi:10.1016/j.cell.2019.05.031 31178118 PMC6687398

[24] Yu G , Wang LG , Han Y , He QY . clusterProfiler: an R package for comparing biological themes among gene clusters. Omics. 2012;16(5):284‐287. doi:10.1089/omi.2011.0118 22455463 PMC3339379

[25] Subramanian A , Tamayo P , Mootha VK , et al. Gene set enrichment analysis: a knowledge‐based approach for interpreting genome‐wide expression profiles. Proc Natl Acad Sci U S A. 2005;102(43):15545‐15550. doi:10.1073/pnas.0506580102 16199517 PMC1239896

[26] F R , H X , P Q , et al.—Single‐cell transcriptomics reveals male germ cells and Sertoli cells. *D ‐ 101630250*. (‐ 2296‐634X (Print)):‐ 944325. 10.3389/fcell.2022.944325 PMC9355508 35938151

[27] Jin S , Guerrero‐Juarez CF , Zhang L , et al. Inference and analysis of cell‐cell communication using CellChat. Nat Commun. 2021;12(1):1088. doi:10.1038/s41467-021-21246-9 33597522 PMC7889871

[28] Aibar S , González‐Blas CB , Moerman T , et al. SCENIC: single‐cell regulatory network inference and clustering. Nat Methods. 2017;14(11):1083‐1086. doi:10.1038/nmeth.4463 28991892 PMC5937676

[29] Johnsen SG . Testicular biopsy score count–a method for registration of spermatogenesis in human testes: normal values and results in 335 hypogonadal males. Hormones. 1970;1(1):2‐25. doi:10.1159/000178170 5527187

[30] Li X , Wang Z , Jiang Z , et al. Regulation of seminiferous tubule‐associated stem Leydig cells in adult rat testes. Proc Natl Acad Sci U S A. 2016;113(10):2666‐2671. doi:10.1073/pnas.1519395113 26929346 PMC4790979

[31] Love MI , Huber W , Anders S . Moderated estimation of fold change and dispersion for RNA‐seq data with DESeq2. Genome Biol. 2014;15(12):550. doi:10.1186/s13059-014-0550-8 25516281 PMC4302049

[32] Schaum N , Lehallier B , Hahn O , et al. Ageing hallmarks exhibit organ‐specific temporal signatures. Nature. 2020;583(7817):596‐602. doi:10.1038/s41586-020-2499-y 32669715 PMC7757734

[33] Flurkey K , Currer JM , Harrison DE . Chapter 20 ‐ Mouse models in aging research. In: Fox JG , Davisson MT , Quimby FW , Barthold SW , Newcomer CE , Smith AL , eds. The Mouse in Biomedical Research. 2nd ed. Academic Press; 2007:637‐672.

[34] Shen X , Wang C , Zhou X , et al. Nonlinear dynamics of multi‐omics profiles during human aging. Nat Aging. 2024;4(11):1619‐1634. doi:10.1038/s43587-024-00692-2 39143318 PMC11564093

[35] Huang Y , Li X , Sun X , et al. Anatomical transcriptome atlas of the male mouse reproductive system during aging. Front Cell Dev Biol. 2021;9:782824. doi:10.3389/fcell.2021.782824 35211476 PMC8861499

[36] Nes WD , Lukyanenko YO , Jia ZH , et al. Identification of the lipophilic factor produced by macrophages that stimulates steroidogenesis. Endocrinology. 2000;141(3):953‐958. doi:10.1210/endo.141.3.7350 10698170

[37] Wiley CD , Flynn JM , Morrissey C , et al. Analysis of individual cells identifies cell‐to‐cell variability following induction of cellular senescence. Aging Cell. 2017;16(5):1043‐1050. doi:10.1111/acel.12632 28699239 PMC5595671

[38] Hernandez‐Segura A , de Jong TV , Melov S , Guryev V , Campisi J , Demaria M . Unmasking transcriptional heterogeneity in senescent cells. Curr Biol. 2017;27(17):2652‐2660.e4. doi:10.1016/j.cub.2017.07.033 28844647 PMC5788810

[39] Mularoni V , Esposito V , Di Persio S , et al. Age‐related changes in human Leydig cell status. Hum Reprod. 2020;35(12):2663‐2676. doi:10.1093/humrep/deaa271 33094328

[40] Lavin Y , Winter D , Blecher‐Gonen R , et al. Tissue‐resident macrophage enhancer landscapes are shaped by the local microenvironment. Cell. 2014;159(6):1312‐1326. doi:10.1016/j.cell.2014.11.018 25480296 PMC4437213

[41] Jung SH , Hwang BH , Shin S , et al. Spatiotemporal dynamics of macrophage heterogeneity and a potential function of Trem2(hi) macrophages in infarcted hearts. Nat Commun. 2022;13(1):4580. doi:10.1038/s41467-022-32284-2 35933399 PMC9357004

[42] Hu Y , Cai TT , Yan RN , Liu BL , Ding B , Ma JH . Single‐Cell RNA sequencing analysis of steroidogenesis and spermatogenesis impairment in the testis of db/db mice. Int J Endocrinol. 2024;2024:8797972. doi:10.1155/2024/8797972 38817616 PMC11139535

[43] Olaniru OE , Kadolsky U , Kannambath S , et al. Single‐cell transcriptomic and spatial landscapes of the developing human pancreas. Cell Metab. 2023;35(1):184‐199.e5. doi:10.1016/j.cmet.2022.11.009 36513063

[44] López‐Otín C , Blasco MA , Partridge L , Serrano M , Kroemer G . Hallmarks of aging: an expanding universe. Cell. 2023;186(2):243‐278. doi:10.1016/j.cell.2022.11.001 36599349

[45] Ma S , Sun S , Geng L , et al. Caloric restriction reprograms the single‐cell transcriptional landscape of *Rattus norvegicus* aging. Cell. 2020;180(5):984‐1001.e22. doi:10.1016/j.cell.2020.02.008 32109414

[46] Li X , Li Y , Jin Y , et al. Transcriptional and epigenetic decoding of the microglial aging process. Nat Aging. 2023;3(10):1288‐1311. doi:10.1038/s43587-023-00479-x 37697166 PMC10570141

[47] Mantovani A , Sica A , Sozzani S , Allavena P , Vecchi A , Locati M . The chemokine system in diverse forms of macrophage activation and polarization. Trends Immunol. 2004;25(12):677‐686. doi:10.1016/j.it.2004.09.015 15530839

[48] van Deursen JM . The role of senescent cells in ageing. Nature. 2014;509(7501):439‐446. doi:10.1038/nature13193 24848057 PMC4214092

[49] Matzkin ME , Calandra RS , Rossi SP , Bartke A , Frungieri MB . Hallmarks of testicular aging: the challenge of anti‐inflammatory and antioxidant therapies using natural and/or pharmacological compounds to improve the physiopathological status of the aged male gonad. Cells. 2021;10(11):3114. doi:10.3390/cells10113114 34831334 PMC8619877

[50] Franceschi C , Bonafè M , Valensin S , et al. Inflamm‐aging. An evolutionary perspective on immunosenescence. Ann N Y Acad Sci. 2000;908:244‐254. doi:10.1111/j.1749-6632.2000.tb06651.x 10911963

[51] Ma S , Ji Z , Zhang B , et al. Spatial transcriptomic landscape unveils immunoglobin‐associated senescence as a hallmark of aging. Cell. 2024;187(24):7025‐7044.e34. doi:10.1016/j.cell.2024.10.019 39500323

[52] K X , P L , J Y , et al.—Single‐cell RNA sequencing reveals transcriptomic landscape and potential targets. *D ‐ 8701199*. (‐ 1460‐2350 (Electronic)):‐ 2189‐2209. 10.1093/humrep/deae199 PMC11447013 39241251

[53] Lokka E , Lintukorpi L , Cisneros‐Montalvo S , et al. Generation, localization and functions of macrophages during the development of testis. Nat Commun. 2020;11(1):4375. doi:10.1038/s41467-020-18206-0 32873797 PMC7463013

[54] Jiao H , Walczak BE , Lee MS , Lemieux ME , Li WJ . GATA6 regulates aging of human mesenchymal stem/stromal cells. Stem Cells. 2021;39(1):62‐77. doi:10.1002/stem.3297 33252174 PMC7772271

