# Supplementary material for: Spatiotemporal mapping reveals Ccl8hi macrophages as key drivers of testicular inflammaging
Source: Clin Transl Med. 2025 Nov 17;15(11):e70527. doi: 10.1002/ctm2.70527 (PMC12623838; doi:10.1002/ctm2.70527)
Supplement: Supplementary file 1 — Supporting Information [file CTM2-15-e70527-s002.docx]

### Supplemental material

Supplemental Figure 1-4.


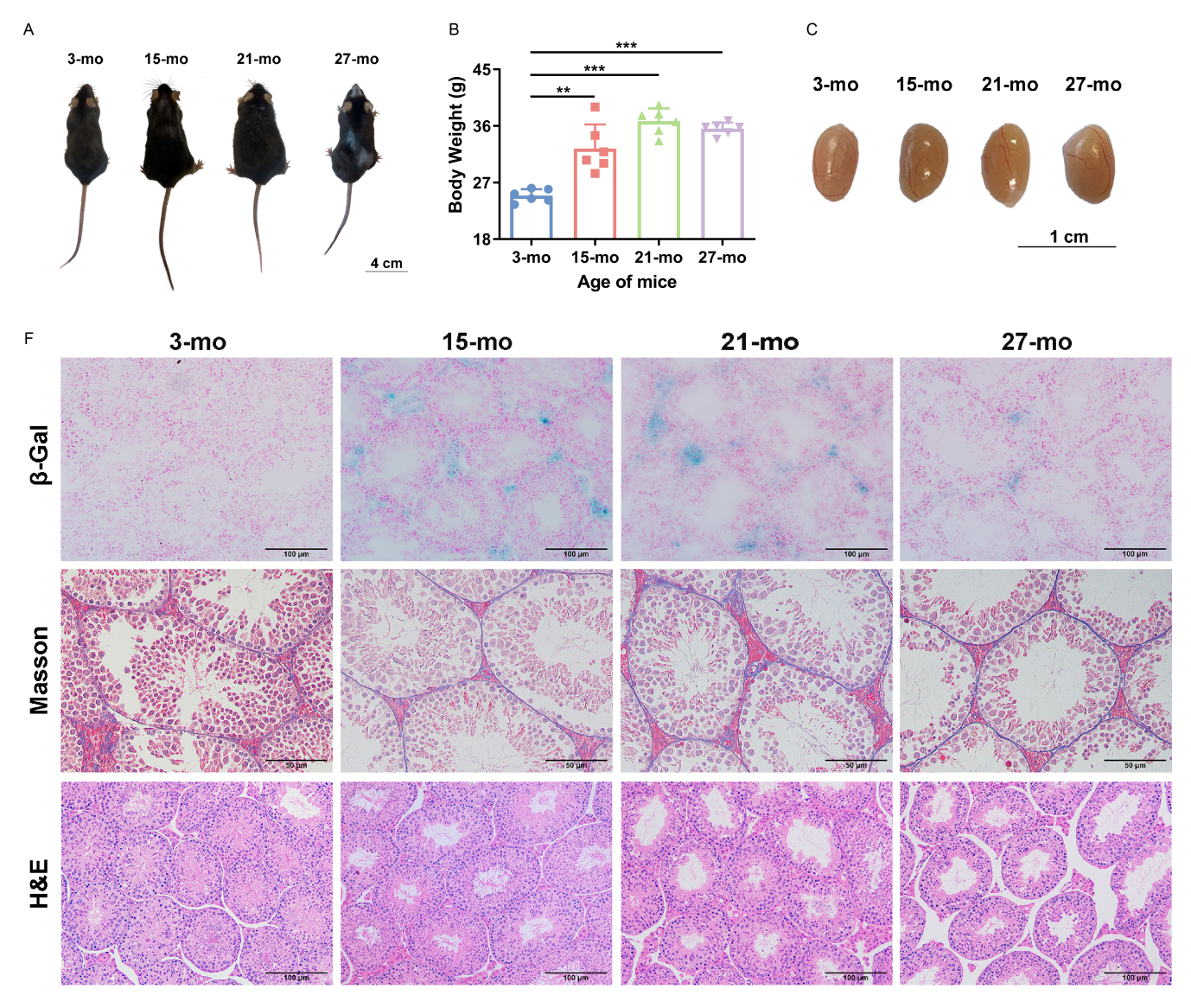


### Supplemental Figure 1. Cellular and molecular characteristics of age-related testicular dysfunction.

(A) Morphological images of mice at 3, 15, 21, and 27 months of age corresponding to human aging (scale bar = 4 cm). (B) Bar plots of mouse body weight at 3, 15, 21, and 27 months of age. (C) Representative images of mouse testes at 3, 15, 21, and 27 months of age. Scale bar, 1 cm. (D) Representative images of β-galactosidase staining, Masson’s Trichrome staining, and H&E staining in cross-sections of the testis from mice at 3, 15, 21, and 27 months of age.


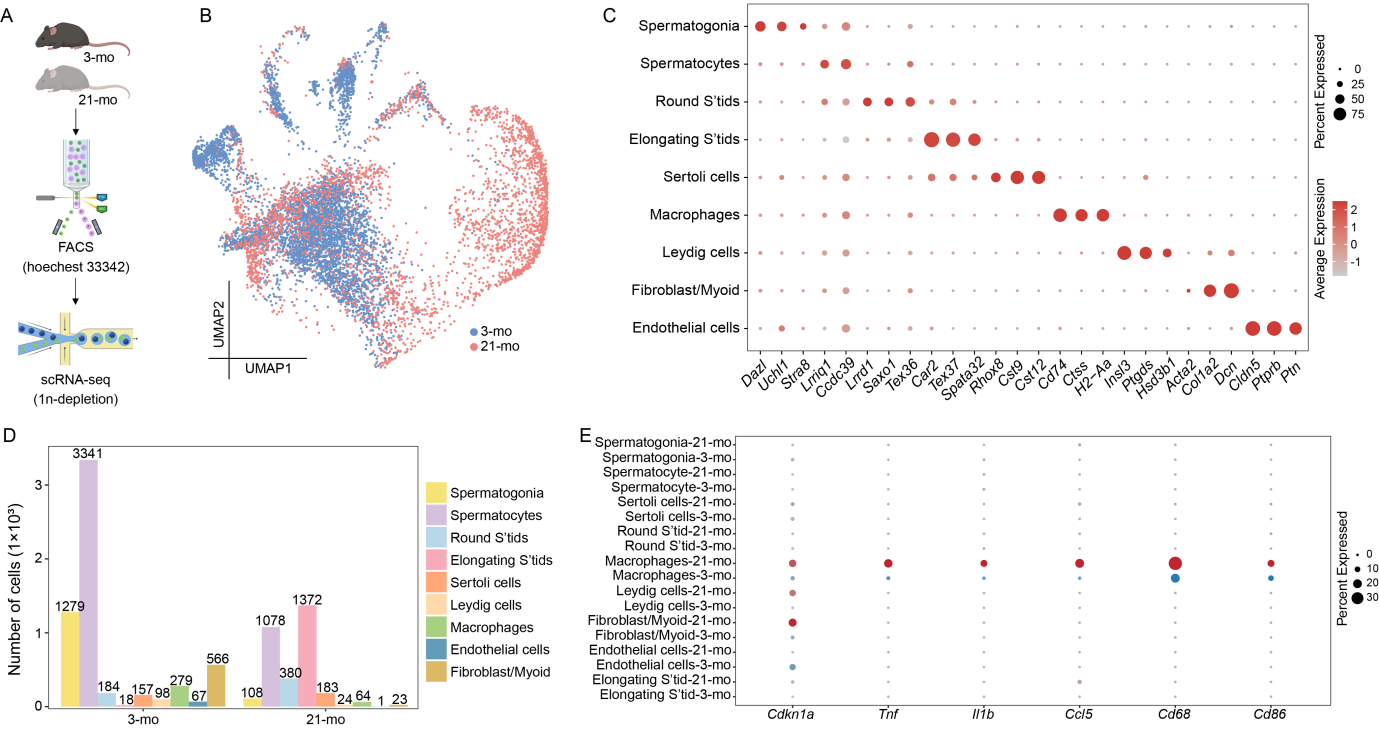


### Supplemental Figure 2. Characterization of TMs by single-cell RNA sequencing. (A) Workflow of scRNA-seq using 1n-depletion flow cytometry approach. (B) UMAP plot of scRNA-seq (1n-depletion) showing testicular cell clusters of 3- and 21-month-old mice. (C) Dot plot showing the expression of representative marker genes in the indicated cell populations. Dot size indicates the fraction of cells within the subpopulation expressing a given marker, color scale, and the average gene expression level. (D) Bar plots showing the number of main somatic cells in young and old mouse testes from scRNA-seq data. (E) Dot plot illustrating the expression of aging-related genes in TMs.


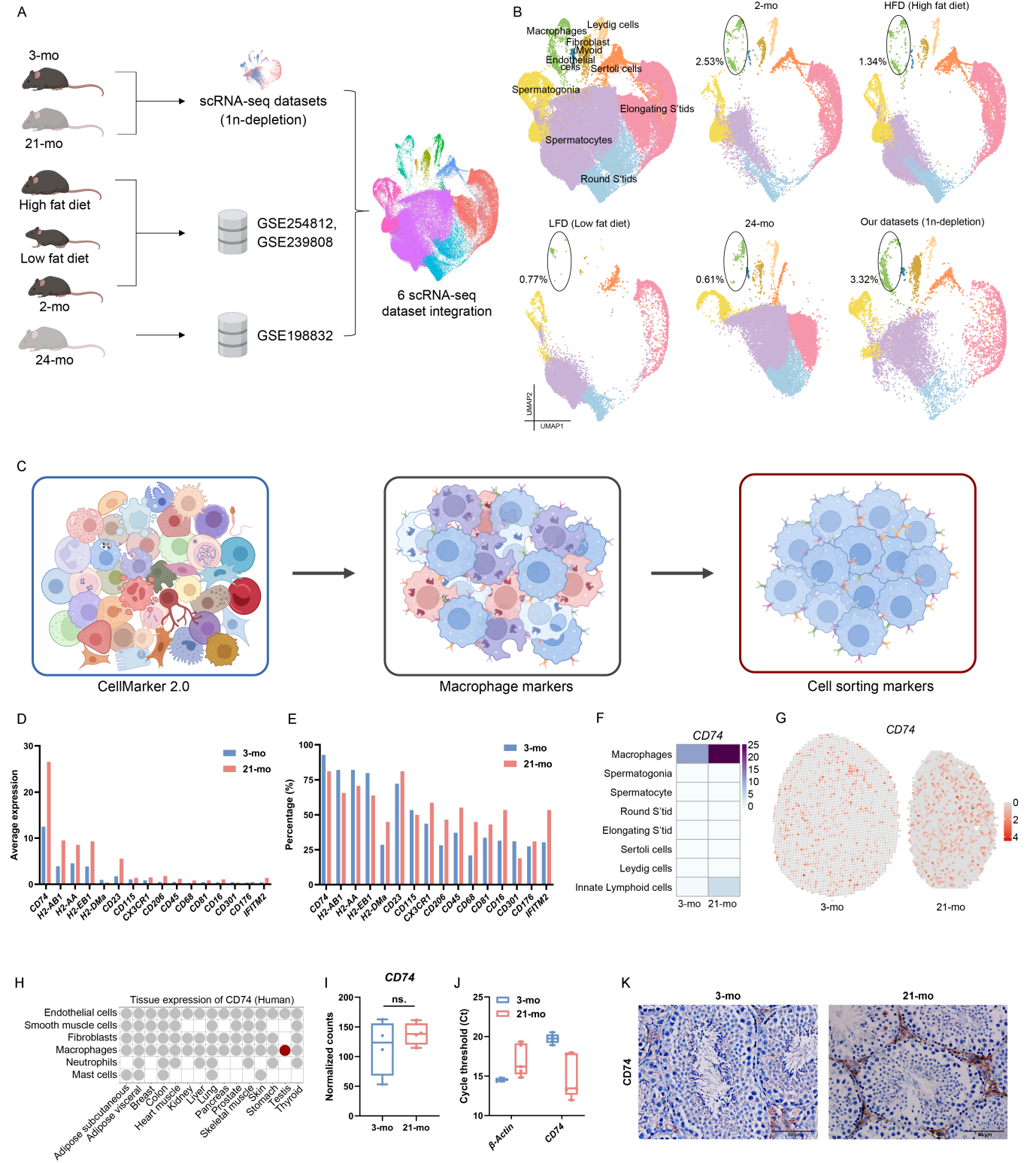


**Supplemental Figure 3. Refined sorting strategy to enrich TMs for single-cell high-resolution profiling.** (A) Integration of multiple single-cell RNA-seq data from mouse testes from GEO datasets (GSE254812, GSE239808, and GSE198832) and our datasets. (B) UMAP of analyzed cells with TMs highlighted for each database separately. (C) Flowchart for screening surface markers of TMs. (D) Bar plots showing the expression of macrophage surface markers from testicular single-cell sequencing data. (E) Proportion of macrophage surface markers in all testicular macrophage cells from scRNA-seq data. (F) Heatmap of CD74 expression in all cell types identified by scRNA-seq. (G) Spatial projection of CD74 on testicular tissue slides of 3 and 21-month-old mice. (H) The relative transcriptomic expression of CD74 in each cell type is based on data from the HPA dataset. (I) CD74 gene expression in the testicular bulk RNA-seq data. (J) qPCR expression of CD74 and β-Actin (n = 6). (K) IHC of CD74 in the testicular tissue from 3- and 21-month-old mice.


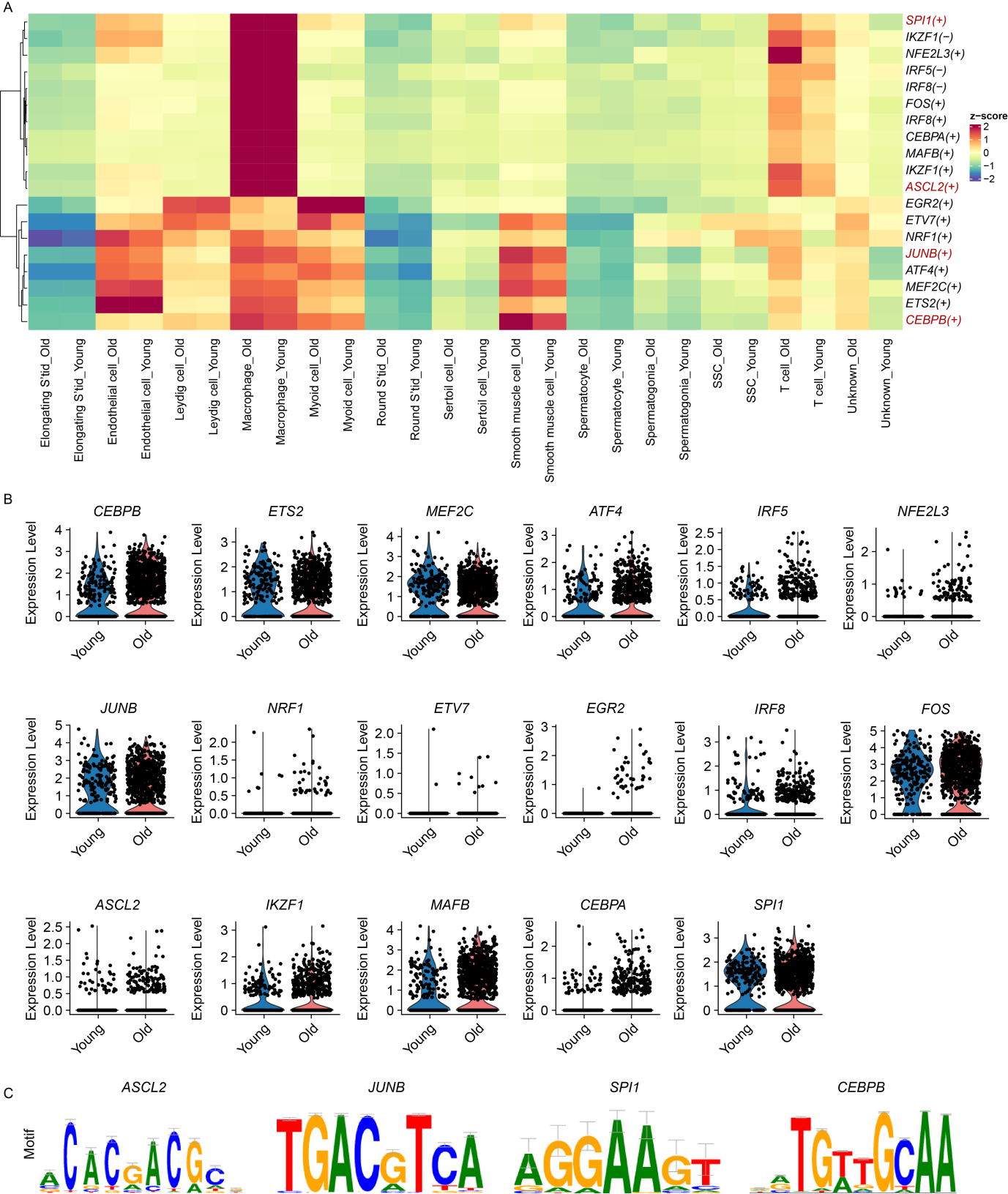


### Supplemental Figure 4. Predicted key TFs in the human TM aging process. (A) Heatmap showing putative TFs of shared DEGs in Figure 6G of human and mouse aging. (B) TF expression in human TMs. (C) TF motifs significantly enriched in human TMs in old age.
