# Supplementary material for: Spatiotemporal mapping reveals Ccl8hi macrophages as key drivers of testicular inflammaging
Source: Clin Transl Med. 2025 Nov 17;15(11):e70527. doi: 10.1002/ctm2.70527 (PMC12623838; doi:10.1002/ctm2.70527)
Supplement: Supplementary file 2 — Supporting Information [file CTM2-15-e70527-s001.docx]

**Table S1. List of primers**

| **Gene** |  | **sequence (5'-3')** |
| --- | --- | --- |
| β-Actin | Forward | TGCTGTCCCTGTATGCCTCT |
| β-Actin | Reverse | TTTGATGTCACGCACGATTT |
| Cd68 | Forward | TCTCTAAGGCTACAGGCTGCTCAG |
| Cd68 | Reverse | GGCTGGTAGGTTGATTGTCGTCTG |
| Cd86 | Forward | TCTGCCGTGCCCATTTACAAAGG |
| Cd86 | Reverse | AAGTTGGCGATCACTGACAGTTCTG |
| Cd163 | Forward | TGGACTGTGGCGTGGCAATTAAC |
| Cd163 | Reverse | TTCTTTGTGGGCTTCGTTGGTCAG |
| Cd206 | Forward | CGCTCTAAGTGCCATCTCAGTTCAG |
| Cd206 | Reverse | TGCCCTTGATTCCAAAGAGTGTGTC |
| Ccl8 | Forward | TGCTTCTTTGCCTGCTGCTCATAG |
| Ccl8 | Reverse | CCATGTACTCACTGACCCACTTCTG |
| Cd74 | Forward | TGTCTCTGTCCTGGTGGCTCTG |
| Cd74 | Reverse | GACGCATCAGCAAGGGAGTAGC |
